# Blockage of CacyBP inhibits macrophage recruitment and improves anti-PD-1 therapy in hepatocellular carcinoma

**DOI:** 10.1186/s13046-023-02885-w

**Published:** 2023-11-16

**Authors:** Jialiang Wang, Xiaoyu Zhang, Xinyi Ma, Dongmei Chen, Meina Cai, Lexin Xiao, Jing Li, Zexuan Huang, Yuehua Huang, Yifan Lian

**Affiliations:** 1 Guangdong Provincial Key Laboratory of Liver Disease Research, the Third Affiliated Hospital of Sun Yat-Sen University, 600 Tianhe Rd., Guangzhou, 510630 China; 2 Department of Infectious Diseases, the Third Affiliated Hospital of Sun Yat-Sen University, 600 Tianhe Rd., Guangzhou, 510630 China

**Keywords:** CacyBP, Myd88, Tumor-associated macrophage, Programmed cell death-1, Hepatocellular carcinoma

## Abstract

**Background:**

Despite remarkable advancements in cancer immunotherapy, the overall response rate to anti-programmed cell death-1 (anti-PD-1) therapy in hepatocellular carcinoma (HCC) patients remains low. Our previous study has demonstrated the critical role of CacyBP/SIP (Calcyclin-Binding Protein and Siah-1 Interacting Protein) as a regulator of HCC development and progression. However, the possible impact of CacyBP on the tumor immune microenvironment has not yet been clarified.

**Methods:**

The expressions of CacyBP and Myd88 in HCC cell lines and tissues was detected by bioinformatics analysis, real-time quantitative PCR, western blotting and immunohistochemistry. The interaction between CacyBP and Myd88 was measured using co-immunoprecipitation and immunofluorescence. In vitro and in vivo assays were used to investigate the regulation of CacyBP on tumor-associated macrophages (TAMs).

**Results:**

We identified that CacyBP was positively correlated with Myd88, a master regulator of innate immunity, and Myd88 was a novel binding substrate downstream of CacyBP in HCC. Additionally, CacyBP protected Myd88 from Siah-1-mediated proteasome-dependent degradation by competitively binding to its Toll/interleukin-1 receptor (TIR) domain. Inhibition of CacyBP-Myd88 signaling subsequently diminished HDAC1-mediated H3K9ac and H3K27ac modifications on the CX3CL1 promoter and reduced its transcription and secretion in HCC cells. Moreover, by using in vitro and in vivo strategies, we demonstrated that depletion of CacyBP impaired the infiltration of TAMs and the immunosuppressive state of the tumor microenvironment, further sensitizing HCC-bearing anti-PD-1 therapy.

**Conclusions:**

Our findings suggest that targeting CacyBP may be a novel treatment strategy for improving the efficacy of anti-PD-1 immunotherapy in HCC.

**Supplementary Information:**

The online version contains supplementary material available at 10.1186/s13046-023-02885-w.

## Introduction

Accompanied by the high incidence and mortality, hepatocellular carcinoma (HCC) is still a threatening health challenge worldwide [1]. According to the global cancer statistics, there were approximately 905 700 new cases and 830 200 deaths of HCC in 2020, among which, patients in China accounted for 50%-55% of the cases [2]. Due to the occult pathogenesis and rapid progression, a considerable proportion of patients with HCC are often diagnosed at advanced stages, therefore not suitable for curative surgery [3]. In fact, systemic molecular agents have been the main therapeutic method for advanced-stage HCC, and the first-line drugs such as lenvatinib [4] and sorafenib [5] have been confirmed to improve the median overall survival (OS) of HCC patients by 11–14 months [6]. However, the curative effect of these drugs is still limited by the occurrence of primary and secondary resistance.

With the rapid development of molecular biology and immunology, the emergence of cancer immunotherapy has revolutionized the management of various advanced tumors. Immune-checkpoint inhibitors (ICIs), especially programmed cell death protein-1 (PD-1) and programmed death-ligand 1 (PD-L1) checkpoint blockade, have been a promising anticancer treatment modalities. ICIs prevent the inactivation of T cells in the tumor microenvironment (TME) to normalize immune responses by blocking the interaction between PD-1 and PD-L1 [7]. Although ICIs have achieved efficacy in certain cancer treatments, two clinical trials showed that only approximately 15–20% of HCC patients showed a durable response to anti-PD-1 monotherapy [7, 8]. Therefore, there is an urgent need to elucidate the underlying mechanism of HCC resistance to ICIs for improving clinical outcomes.

Tumor-associated macrophages (TAMs) are among the most abundant immune cells in the HCC microenvironment [9–11]. In response to distinct signals, TAMs can be recruited and activated to two polarization states in HCC. M1 macrophages typically play a proinflammatory role, while M2 macrophages usually tend to exert the an anti-inflammatory function [12, 13]. Accumulating evidences have shown that high density of TAMs is associated with poor prognosis in many cancers, including HCC [14]. Generally, TAMs are guided into the tumor milieu and subsequently educated to establish an immunosuppressive TME by tumor cell-derived extracellular vesicles and cytokines which are essential for cell-to-cell communication [15, 16]. In turn, these infiltrated-macrophages can also affect tumor cells by releasing cytokines or directly interacting, ultimately leading to immune escape and tumor progression [15, 17–19]. Thus, blocking TAM recruitment and infiltration may be a promising strategy for cancer treatment.

CacyBP/SIP (Calcyclin-Binding Protein and Siah-1 Interacting Protein) is a multiligand protein which was first identified in Ehrlich ascites tumor cells [20]. CacyBP is widely expressed in different mammalian cells, and participates in a broad array of biological processes by binding with several target proteins. It has been reported that CacyBP interacts with cytoskeletal proteins such as tubulin, actin and tropomyosin, to regulate the dynamics of cytoskeleton [21, 22]. Additionally, CacyBP forms an SKP1, CUL1, F-box protein (SCF) complex with Siah-1 and TBL1 to promote the ubiquitination and degradation of nonphosphorylated β-catenin [23, 24]. CacyBP can also serve as a phosphatase, blocking the MAPK signaling cascade by binding and dephosphorylating ERK1/2 and P38 [25, 26]. Although literatures have revealed overexpression of CacyBP in many cancers and its association with poor prognosis [26–33], the impact of CacyBP expression on the immune microenvironment, especially on regulating TAMs, remains poorly defined.

Here, by using western blotting and immunohistochemistry, we demonstrated a positive correlation between the expression of CacyBP and Myd88, a central modulator in innate immune signaling, in HCC tissues. Particularly, we found that CacyBP protected Myd88 from ubiquitination and proteasomal degradation by disrupting its interaction with the E3 ligase Siah-1. In addition, inhibition of CacyBP-Myd88 axis impaired the recruitment of TAMs into the TME by reducing CX3CL1 secretion and sensitized HCC-bearing mice to anti-PD-1 therapy. Our findings provide a novel insight into the role of CacyBP in TME regulation and offer a potential strategy for HCC immunotherapy.

## Materials and methods

### Patient samples

We obtained a cohort of 139 HCC patients who underwent primary resection at Sun Yat-sen University Cancer Center (Guangzhou, China) for prognosis analysis and correlation analysis. To compare Siah-1 expression between HCC tissues and adjacent nontumor tissues, another 34 fresh paraffin-embedded HCC specimens were obtained from the Third Affiliated Hospital of Sun Yat-sen University (Guangzhou, China). Surgical tumor resection was performed on each patient in the Department of Hepatobiliary Surgery. HCC tissues were cut into the proper size and fixed in 4% paraformaldehyde for immunohistochemistry. The study was approved by the Institute Research Ethics Committee at the Sun Yat-sen University Cancer Center and the Third Affiliated Hospital of Sun Yat-sen University (ethics numbers: [2018]02–033-01). Written informed consent was obtained from each patient.

### Animal experiments

C57BL/6 mice (6 weeks, male) were purchased from GemPharmatech (Jiangsu, China). All mice were handled according to the Guide for the Care and Use of Laboratory Animals. The procedures were approved by the Institutional Animal Care and Use Committee of the Third Affiliated Hospital of Sun Yat-sen University (ethics numbers: 2023F207).

For subcutaneous tumor xenograft models, all C57BL/6 mice were randomly divided into 4 groups. A total of 2 × 10^6^ Hepa1-6 cells(shNC or shCacyBP) were injected into the hind flanks of the mice. Tumors were allowed to grow for 7 days and then the mice were administrated with PBS or anti-PD-1 antibody(Bio X Cell, 200 μg/mouse, intraperitoneal injection) every 3 days. Tumor growth was monitored every 3 days, and the mice were euthanized after 13 days post-injection via cervical dislocation. All tumors from each group were extracted and weighed. The tumor volume was measured using the following formula: 0.5 × (larger diameter) × (smaller diameter)^2^.

For orthotopic tumor xenograft models, C57BL/6 mice were randomly divided into 2 groups. A total of 1 × 10^6^ shNC or shCacyBP Hepa1-6 cells(1 × 10^6^ cells/mouse) were mixed with Matrigel matrix(354234, Corning) and orthotopically injected into the hepatic capsule of the right liver lobe. After 7 days, the mice were euthanized by cervical dislocation, and livers were collected for histological and flow cytometric analyses.

### Reagents and antibodies

Commercially available reagents and antibodies used in this study are listed as follows: hIL-1β (8900, Cell Signaling Technology), hCX3CL1 (30–31-20, Proteintech), PMA (16561–29-8, Sigma-Aldrich), MG132 (474,790, Calbiochem), CHX (C7698, Sigma-Aldrich), in vivo PD-1 antibody (BE0146, BioXcell), anti-CacyBP (11745–1-AP, Proteintech), anti-Myd88 (4283, Cell Signaling Technology), anti-Myd88 (SC74532, Santa Cruz, for immunofluorescence only), anti-Siah-1 (GTX55799, GeneTex), anti-Myc-tag (2276, Cell Signaling Technology), anti-HA-tag (3724, Cell Signaling Technology), anti-Flag-tag (F3165, Sigma-Aldrich), anti-Tubulin (66031, Proteintech), anti-HDAC1 (34589, Cell Signaling Technology, for ChIP assay), anti-HDAC1 (5356, Cell Signaling Technology, for western blotting), anti-HDAC2 (5113, Cell Signaling Technology), anti-HDAC3 (3949, Cell Signaling Technology), anti-HDAC4 (7628, Cell Signaling Technology), anti-HDAC6 (7558, Cell Signaling Technology), anti-Lamin B1 (66095–1, Proteintech), anti-β-actin (66009–1, Proteintech), anti-GAPDH (60004–1, Proteintech), anti-H3K9ac (9649, Cell Signaling Technology), anti-H3K27ac (8173, Cell Signaling Technology), anti-CD68 (ab182422, Abcam), anti-CD206 (ab64693, Abcam), anti-CD206 (141704, Biolegend, for flow cytometry only), anti-CD4 (25229, Cell Signaling Technology), anti-CD8 (98941, Cell Signaling Technology), anti-F4/80 (123137, Biolegend), anti-CD163 (ab182422, Abcam), anti-phospho-p65(Ser536) (3033, Cell Signaling Technology), anti-CX3CL1 (MAB3651, RD), anti-Arg-1 (12–3697-82, Invitrogen), anti-CD11b (101212, Biolegend), anti-Ly6c (128024, Biolegend). All other chemical reagents were obtained from Sigma-Aldrich, unless otherwise indicated.

### Cell culture

Human HCC cell lines, PLC/PRF/5 and Huh7, human embryonic kidney cell line, HEK293T, human leukemia monocytic cell line, THP-1 and murine hepatoma cell line from a C57L mouse, hepa1-6, were purchased from Cellcook Company (Guangzhou, China). All cell lines were cultured in Dulbecco’s modified Eagle’s medium (DMEM) or RPMI-1640 medium containing 10% fetal bovine serum (FBS) in a humidified incubator with 5% CO_2_ at 37 °C. All cell lines were thawed from early passage stocks and passaged every 2 days.

### Plasmid construction and transfection

The complementary DNA (cDNA) encoding full-length human *CACYBP*, *SIAH1* and *MYD88* genes were amplified using PCR from a cDNA library of HEK293T cells and subcloned into the pcDNA3.1( +) vector (Invitrogen). During PCR, a sequence encoding a Flag tag (GATTACAAGGATGACGACGATAAG) or a HA tag (TACCCATACGATGTTCCAGATTACGCT) was added to the C-terminus of Myd88 and Siah-1 or the N-terminus of CacyBP, respectively. Serial Myd88 truncation mutants were generated by deleting the corresponding protein domains: △TIR, deleting amino acids 159 to 296; △INT, deleting amino acids 117 to 155; and △DD, deleting amino acids 54 to 110. Correct constructs were all confirmed using DNA sequencing. Transient transfection was performed using ViaFect Transfection Reagent (Promega) following the manufacturer’s suggested procedures.

### RNA isolation and real-time quantitative PCR (qRT-PCR)

Total RNA from cultured cells were isolated with TRIzol reagent (Sigma-Aldrich) and 2 μg of purified RNA was reverse-transcribed using the GoScript Reverse Transcription System (Promega). qRT-PCR was performed with Platinum SYBR Green (Invitrogen) according to the manufacturer’s instructions on a LightCycler 480 PCR platform (Roche). The relative gene expression was determined based on the 2^−ΔΔCt^ method.

The sequences of primers used are as follows: *CACYBP* (forward: 5’-CTCCCATTACAACGGGCTATAC-3’, reverse: 5’-GAACTGCCTTCCACAGAGATG-3’)*, MYD88* (forward: 5’-GGCTGCTCTCAACATGCGA-3’, reverse: 5’-CTGTGTCCGCACGTTCAAGA-3’)*, IL6* (forward: 5’-ACTCACCTCTTCAGAACGAATTG-3’, reverse: 5’-CCATCTTTGGAAGGTTCAGGTTG-3’)*, CXCL8* (forward: 5’-TTTTGCCAAGGAGTGCTAAAGA-3’, reverse: 5’-AACCCTCTGCACCCAGTTTTC-3’)*, TNF* (forward: 5’-CCTCTCTCTAATCAGCCCTCTG-3’, reverse: 5’-GAGGACCTGGGAGTAGATGAG-3’)*, CX3CL1* (forward: 5’-ACCACGGTGTGACGAAATG-3’, reverse: 5’-TGTTGATAGTGGATGAGCAAAGC-3’)*, CCL14* (forward: 5’-CCAAGCCCGGAATTGTCTTCA-3’, reverse: 5’-GGGTTGGTACAGACGGAATGG-3’)*, CCL15* (forward: 5’-TCCCAGGCCCAGTTCATAAAT-3’, reverse: 5’-TGCTTTGTGAGATGTAGGAGGT-3’)*, CCL16* (forward: 5’-ACAGAAAGGCCCTCAACTGTC-3’, reverse: 5’-TCCTTGATGTACTCTTGGACCC-3’)*, CCL22* (forward: 5’-ATCGCCTACAGACTGCACTC-3’, reverse: 5’-GACGGTAACGGACGTAATCAC-3’)*, PPBP* (forward: 5’-GTAACAGTGCGAGACCACTTC-3’, reverse: 5’-CTTTGCCTTTCGCCAAGTTTC-3’)*, CXCL9* (forward: 5’-CCAGTAGTGAGAAAGGGTCGC-3’, reverse: 5’-AGGGCTTGGGGCAAATTGTT-3’)*, CCL5* (forward: 5’-CCAGCAGTCGTCTTTGTCAC-3’, reverse: 5’-CTCTGGGTTGGCACACACTT-3’)*, HDAC1* (forward: 5’-CTACTACGACGGGGATGTTGG-3’, reverse: 5’-GAGTCATGCGGATTCGGTGAG-3’)*, GAPDH* (forward: 5’-GGAGCGAGATCCCTCCAAAAT-3’, reverse: 5’-GGCTGTTGTCATACTTCTCATGG-3’).

### RNA-Sequencing

Total mRNA was enriched by oligo (dT) beads (Epicentre, USA) and reverse-transcribed into cDNA using random primers. The mRNA was ligated with proprietary 5’ and 3’ adapters. The ligation products were reverse-transcribed by PCR amplification to generate a cDNA library, which was sequenced using an Illumina HiSeq 2500 by Gene Denovo Biotechnology Co. (Guangzhou, China).

### Western blotting

The cells were seeded in 6-well plates and treated as indicated. At approximately 70%-80% confluence, the cells were lysed in RIPA buffer (P0013B, Beyotime) containing protease (4693116001, Roche) and phosphatase (4693116,001, Roche) inhibitor cocktails. The concentration of protein was determined by the BCA sample kit (23225, Pierce). After normalization, the protein was boiled in 3 × SDS loading buffer for 10 min. Then 10–20 Sg of protein was subjected to 10%, 12% or 15% SDS-PAGE, transferred to the PVDF membrane (BioRad Laboratories) and blocked with 5% nonfat milk or BSA (23227, Thermo Fisher). The membranes were incubated with primary antibodies at 4 °C overnight, and subsequently with species-specific HRP-conjugated secondary antibodies (31460, Thermo Fisher) at room temperature for 1 h. Enhanced chemiluminescence (WBKLS0100, Millipore) was used to visualize the bands.

### Cell fractionation assay

For nuclear and cytoplasmic protein extraction, the cells were seeded in a 6-well plate and were treated as indicated. Then the cells were collected and a protein extraction kit (P0027, Beyotime, China) was used to extract cytoplasmic and nuclear proteins according to the manufacturer’s instructions. Briefly, the cells were washed in precooled 1 × PBS and resuspended in the 200 μL cell lysis buffer (10 mM HEPES, pH = 7.9; 10 mM KCl; 0.1 mM EDTA; 1 mM DTT, 0.4% IGEPAL) containing protease inhibitors. Cell lysates were plated on ice for 15 min and were spun in a microcentrifuge at 4 °C and 14000 rpm for 5 min. The supernatants were collected and stored at -80 °C for analysis when needed. The precipitates were washed and 50 μL nuclear extraction buffer (0.4 M NaCl; 20 mM HEPES, pH = 7.9; 1 mM EDTA; 1 mM DTT; 1 mM PMSF) was added with vigorous shaking on ice for 30 min. The nuclear extracts were aliquoted and stored at -80 °C until use.

### Immunohistochemistry

For immunohistochemistry, paraffin-embedded HCC samples were deparaffinized and rehydrated in xylene twice for 15 min and rehydrated by graded ethanol five times for 5 min. After incubation with 3% hydrogen peroxide for 10 min, the sections were boiled in an electric pressure cooker in ethylenediamine tetraacetic acid (EDTA) buffer (pH = 8.0) to retrieve antigen for 2 min. Then the slides were incubated with primary antibodies overnight at 4 °C and secondary antibody (K5007, Dako) at room temperature for 30 min. Signals were detected in freshly prepared DAB substrate solution (K5007, Dako) at room temperature for 5 min and nuclear counterstaining was performed with hematoxylin (C0107, Beyotime) for 1 min. Images were obtained using an automated inverted research microscope (DMI4000, Leica), and each section was evaluated by two independent pathologists blinded to the clinical status of the patients. The area was graded on a scale of 0–4 points (< 25%, 1; 25%-50%, 2; 50%-75%, 3; > 75%, 4) and the intensity was graded on a scale of 0–3 points (no staining, 0; weak staining, 1; moderate staining, 2; strong staining, 3). The product of two values was defined as the final expression score.

### Flow cytometry

For M2 macrophage polarization analysis, 100 nM PMA-treated THP-1 monocytes were seeded in a 6-well plate and coculture with or without HCC cells for 48 h. After that, the cells were washed with cold 1 × PBS and incubated with 0.25 μg anti-CD11b in the dark at room temperature for 30 min. The cells were then centrifuged and washed with 1 × PBS twice. Cell fixation and permeability were performed using FIX & PERM Cell Permeabilization Kit according to the manufacturer’s instructions (GAS004, Invitrogen). After that, the cells were incubated with 1 μg anti-CD206 for intracellular staining. After staining, the cells were washed with cold 1 × PBS twice and analyzed using a Beckman-Coulter CytoFLEX LX Flow Cytometer.

### ELISA

The cells were seeded in a 6-well plate and transfected with the indicated siRNA for 72 h. Cell culture media were removed and replaced with fresh serum-free DMEM for another 24 h. Then, the supernatants were collected and centrifuged at 10,000 rpm for 10 min. The amount of secreted CX3CL1 was detected using specific ELISA kit according to the manufacturer’s instructions (EK1209-96, Multiscience).

### Immunoprecipitation

The cells were harvested in NETN buffer (100 mM NaCl, 20 mM Tris–Cl pH = 8.0, 0.5 mM EDTA, 0.5% IGEPAL) after transfection with plasmids as indicated. After centrifugation at 14,000 rpm for 10 min at 4 °C, the supernatants were incubated with Flag-agarose beads (A2220, Sigma-Aldrich) at 4 °C overnight. After that, the beads were washed five times with NETN buffer and the immunoprecipitates were collected and boiled in 50 μL of 1 × SDS-PAGE loading buffer for 10 min. Finally, the supernatants were analyzed with corresponding antibodies by western blotting.

### Immunofluorescence

Cells cultured on 4-well chamber slides (07–2104, Biologix) were fixed with 4% formaldehyde for 10 min and permeabilized with 0.5% Triton X-100 (QLT-100, Jetway Biotech) for 5 min. The cells were blocked with 5% BSA for 30 min at 37 °C, treated with the indicated primary antibodies at 4 °C overnight and incubated with the secondary antibodies conjugated with Alexa Fluor-488 (A11029, Life Technologies) or -594 (A11005, Life Technologies) for 45 min at room temperature. After washing with 1 × PBS, the cells were stained with 50 ng/ml DAPI (P0131, Beyotime) for 5 min at room temperature and evaluated using a Zeiss LSM710 confocal microscope.

### Ubiquitination assay

The cells were seeded in a 6-well plate and transfected with the indicated plasmids for 36 h. After that, the cells were harvested using denatured buffer (6 M guanidine-HCL, 0.1 M Na_2_HPO_4_, 10 mM imidazole), the cell lysates were incubated with nickel beads (Ni–NTA, R90101, Invitrogen) for 3 h and washed, and the cell extract was analyzed by western blotting.

### Proximity ligation assay (PLA)

PLA was performed using the Duolink In Situ Red Starter Kit Mouse/Rabbit (DUO92004, Millipore) according to the manufacturer’s instructions. In brief, the cells were seeded onto an 8-well chamber slide (07–2108, Biologix) at a density of 1 × 10^4^ cells/well overnight, fixed with 4% paraformaldehyde for 10 min at room temperature, washed with 1 × PBS, and permeabilized with 0.1% Triton X-100 for 5 min. The cells were washed with Wash Buffer A, blocked with Duolink Blocking Buffer for 30 min at room temperature, and then incubated with indicated primary antibodies overnight at 4 °C. The next day, the cells were washed repeatedly in Wash Buffer A and incubated with the appropriate Duolink secondary antibodies for 1 h at 37 °C. The ligation and amplification steps of the PLA were performed according to the manufacturer’s protocol. After final washes with Wash Buffer B at room temperature, slides were mounted with Duolink In Situ Mounting Medium with DAPI. Fluorescence signals were imaged using a Zeiss LSM710 confocal microscope.

### ChIP assay

ChIP assay were analyzed using a Chromatin Immunoprecipitation Assay Kit (17–295, Millipore) according to the manufacturer’s instructions. Briefly, PLC/PRF/5 cells were transfected with plasmids as indicated for 72 h. The cells were cross-linked with 1% formaldehyde (F8775, Sigma-Aldrich) at room temperature for 10 min and then quenched by glycine addition. After washing, the cells were harvested in the lysis buffer at 4 °C for 30 min and sonicated (Sonifier 450D, Branson)(50% amplitude, 10 s pulse, 30 s rest on ice, 4 cycles) to generate DNA fragments (200–1,000 base pairs in length). A total of 10 μg protein-DNA complexes were immunoprecipitated with the indicated antibodies or isotype-matched IgGs. Next, the immunoprecipitated DNA was purified and used for qPCR analysis.

### Statistical analysis

Data were presented as mean ± SD, and the results were analyzed using the GraphPad Prism 7.0 (Dotmatics). A chi-square test was employed to analyze the relationship between CacyBP expression and Myd88 expression. The Kaplan–Meier analysis was employed for the survival analysis. Independent t-test was applied to analyze differences between two groups, and one-way analysis of variance (ANOVA) was used for multiple comparisons. The significance of differences was classified as *p < 0.05, **p < 0.01, ***p < 0.001, or ns (not significant).

## Results

### Myd88 is a binding substrate of CacyBP in HCC

**Fig. 1:**
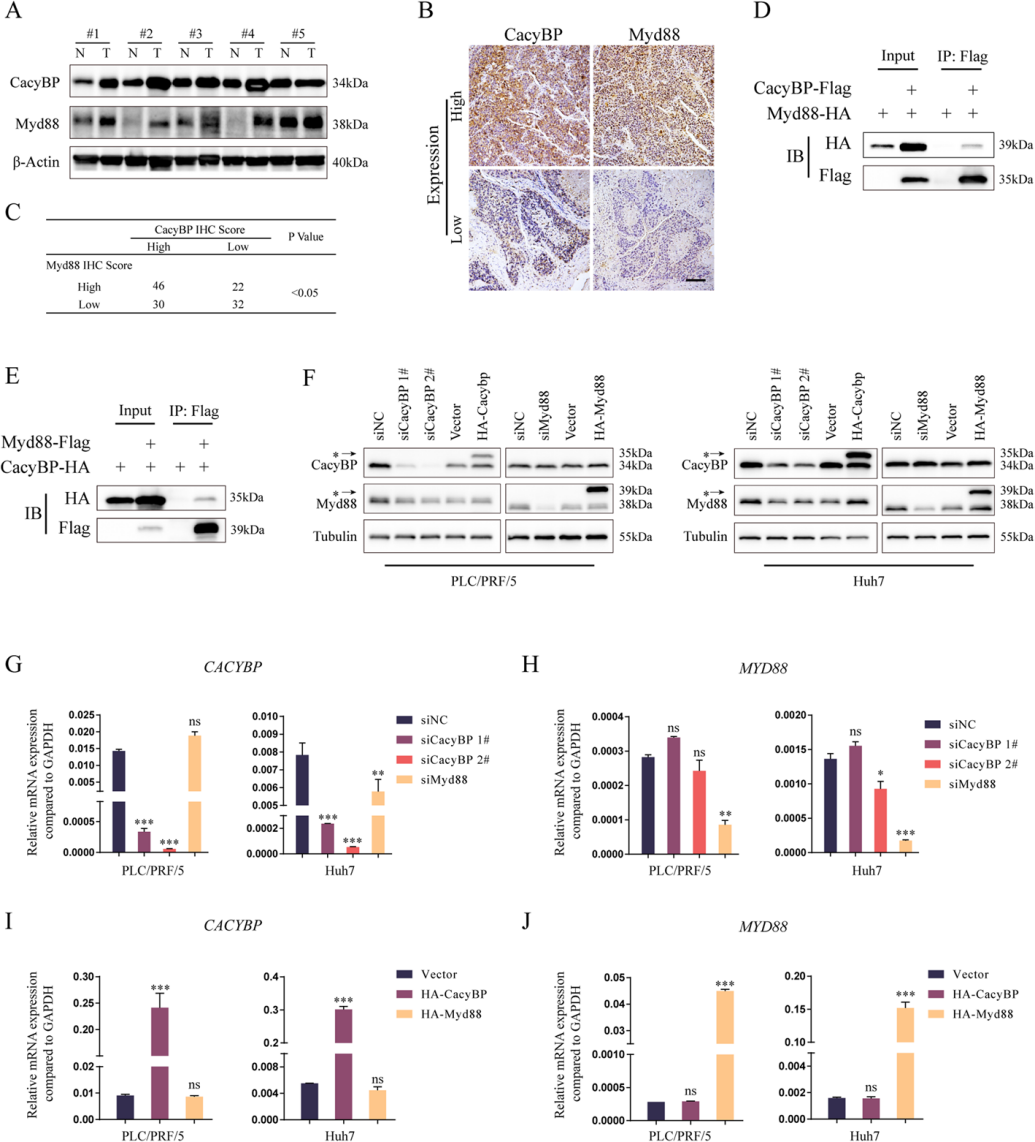
Evaluated CacyBP is correlated with high Myd88 expression in HCC. **A** CacyBP and Myd88 protein levels from 5 pairs of HCC and matched adjacent nontumor tissues were tested by western blotting. **B** Representative immunohistochemistry images of CacyBP and Myd88 expressions in 130 HCC tissues. Bar: 500 μm. **C** Correlation analysis of CacyBP and Myd88 expression scores in the immunohistochemistry assay was performed by χ.^2^-test. **D**-**E** Immunoprecipitation and western blotting experiments were performed using anti-HA agarose on lysates derived from HEK293T cells exogenously expressing Flag-tagged CacyBP and HA-tagged Myd88 (**D**), or Flag-tagged Myd88 and HA-tagged CacyBP (**E**). **F** Protein expression levels of Myd88 and CacyBP were detected by western blotting. Asterisks indicate exogenously expressed proteins. (G-H) Detection of *CACYBP* and *MYD88* mRNA expression in PLC/PRF/5 and Huh7 cells after CacyBP and Myd88 knockdown. **I**-**J** Detection of *CACYBP* and *MYD88* mRNA expression in PLC/PRF/5 and Huh7 cells after CacyBP and Mydd88 overexpression. ns, not significant; *p < 0.05; **p < 0.01; ***p < 0.001

To investigate the potential impact of CacyBP on TME regulation in HCC, we collected 139 HCC clinical specimens and evaluated the expression relationships between CacyBP and the core immune regulator Myd88 by western blotting and immunohistochemistry. As shown in Fig. 1A, both CacyBP and Myd88 were upregulated in HCC tissues as compared to adjacent normal liver tissues. In parallel, χ^2^ analysis showed that patients bearing high CacyBP expression displayed a significant correlation with increased Myd88 (Fig. 1B and C). In coimmunoprecipitation experiments, we observed that exogenously expressed HA-tagged CacyBP or Myd88 could be coprecipitated with Flag-tagged Myd88 or CacyBP in HEK293T cells, respectively (Fig. 1D and E), indicating that the two proteins were able to interact with each other. Moreover, in HCC cells, CacyBP inhibition led to a reduced protein level of Myd88, while overexpressed CacyBP elevated Myd88 protein expression. However, overexpression or knockdown of Myd88 protein did not significantly affect the expression of CacyBP (Fig. 1F). We further found that there was no obvious change in the *CACYBP* or *MYD88* mRNA level after enforced knockdown or overexpression of one another (Fig. 1G-J). Taken together, these observations suggested that Myd88 may be a novel binding substrate downstream of CacyBP.

### CacyBP is involved in Siah-1-mediated degradation of Myd88

**Fig. 2:**
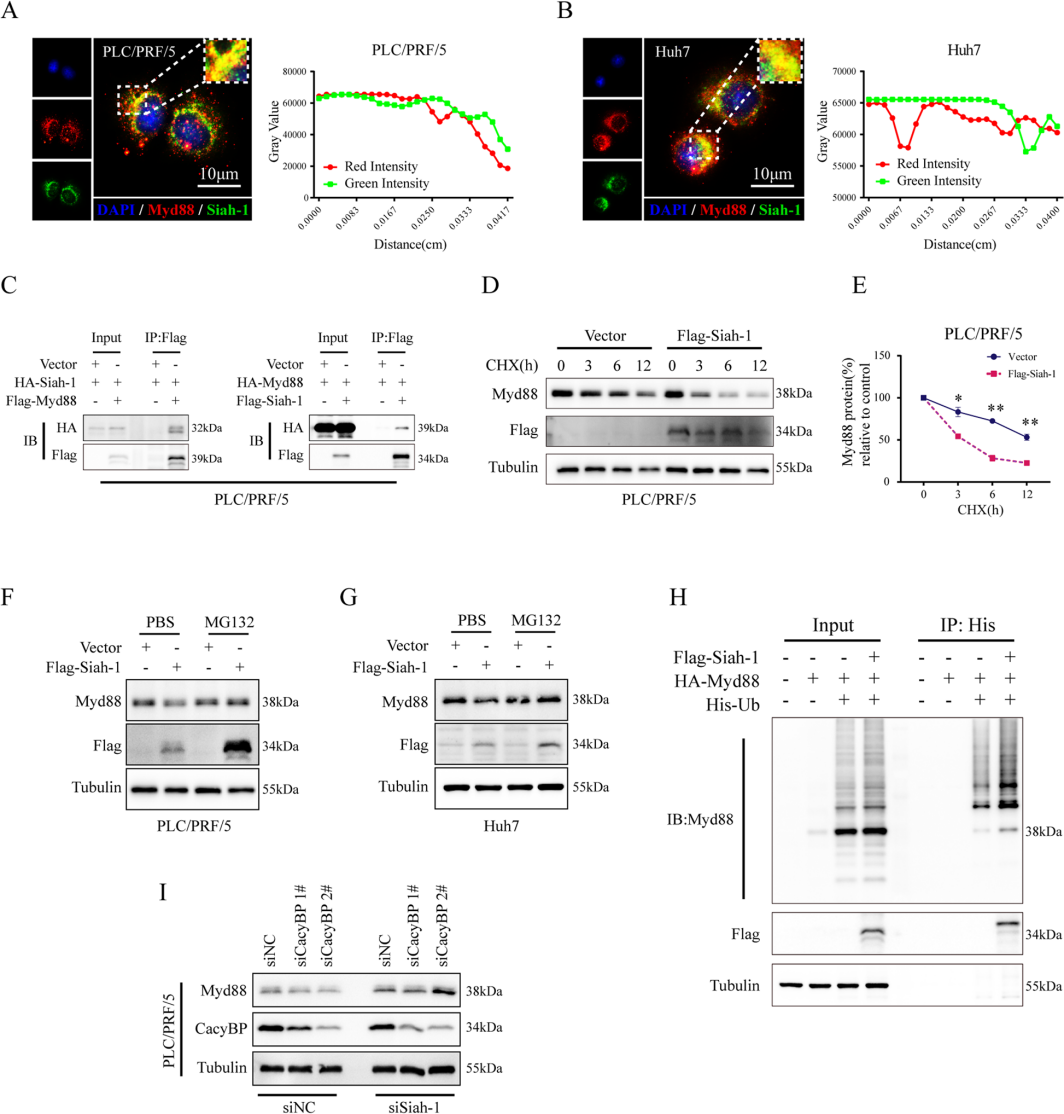
E3 ligase Siah-1 is responsible for CacyBP-mediated Myd88 degradation. **A**-**B** Representative immunofluorescence images and signal quantification show the colocation of Myd88 and Siah-1 in PLC/PRF/5 and Huh7 cells. **C** Coimmunoprecipitation analysis of Siah-1 and Myd88 in HCC cells transfected with the indicated plasmids. **D** CHX chase assay for endogenous Myd88 degradation in PLC/PRF/5 cells. **E** Quantification of Myd88 protein levels in (**D**). **F**-**G** Detection of Myd88 protein expression after Siah-1 overexpression with or without MG132 treatment in HCC cells. **H** Detection of Myd88 polyubiquitination after Siah-1 overexpression in HEK293T cells. **I** Detection of Myd88 protein expression in PLC/PRF/5 cells after CacyBP depletion with or without Siah-1 knockdown. *p < 0.05; **p < 0.01

Previous studies have demonstrated that the ubiquitin–proteasome system plays a major role in regulating Myd88 expression. Given the fact that CacyBP was found to be an adaptor of ubiquitin ligase complexes responsible for the substrate degradation, we speculated that CacyBP may help to recruit Myd88 and facilitate its proteasome-mediated degradation in HCC cells. Therefore, in order to explore the underlying mechanism, we first referred to the BioGrid database [34], a resource dedicated to protein interaction networks, and retrieved 148 and 46 binding candidates for CacyBP and Myd88, respectively (Table 1). The Venn diagram showed that two E3 ubiquitin ligases, namely CYLD and Siah-1, were the common candidates predicted to interact with both CacyBP and Myd88 (Figure S1A). Immunoprecipitation experiments further confirmed that exogenously transfected HA-tagged Siah-1 but not CYLD can bind to CacyBP (Figure S1B and S1C), and overexpression of Siah-1 rather than CYLD could markedly decrease exogenous Myd88 expression in cells (Figure S1D). In addition, in contrast to the tumor-promoting role of CacyBP, Siah-1 expression tended to be downregulated in HCC tissues (Figure S1E and S1F), and patients with reduced Siah-1 expression showed a lower OS and disease-free survival (DFS) (Figure S1G-S1I). Notably, confocal immunofluorescence assays demonstrated that Siah-1 and Myd88 were colocalized in PLC/PRF/5 and Huh7 cells (Fig. 2A and B). The interaction was further confirmed by co-immunoprecipitation (Fig. 2C). Upon cycloheximide (CHX) chase treatment, Siah-1 overexpression significantly reduced the protein half-life of Myd88, while proteasome inhibitor MG132 can abolished Siah-1-induced degradation of Myd88 (Fig. 2D-G). We also observed that Siah-1 could induce the polyubiquitination of Myd88 by in vivo ubiquitin assays (Fig. 2H). Moreover, silencing of Siah-1 effectively reversed the decreased expression of Myd88 after CacyBP depletion in HCC cells (Fig. 2I). Taken together, these results indicated that Siah-1 is an important E3 ligase involved in the process of CacyBP-regulated Myd88 degradation.

**Table 1:** Summary of CacyBP and Myd88 interaction proteins in BioGrid database

	Interaction Proteins
CACYBP	AAR2, AARS, ACO2, ACTR1A, ACTR2, AGR2, AIM2, AKAP1, AKIRIN2, ANAPC2, Apc2, ARHGAP29, ATG16L1, BIRC3, BRD4, CACYBP, CAPZB, CCR2, CEP104, CEP192, CHMP4C, CLUAP1, CTNNB1, CYLD, DDRGK1, DERL1, DHFRL1, DIRAS3, DLD, DNAJB5, DNAJC21, DNAJC7, DUSP13, ECT2, EFTUD2, EGLN3, ERBB2, ESR1, EWSR1, EYA3, FAF1, FAM120C, FAS, FBXO7, FERMT1, FKBP4, FKBP5, FKBP8, FN1, FRMD5, FZR1, GBF1, GLE1, HDAC4, HSD17B10, HSP90AA1, HSP90AB1, HSP90B1, IFI16, IPO11, IRS4, ISG15, ITGA4, KIAA1429, KIF14, LAMTOR1, LCK, LGALS9, LMAN1, LRRC6, LRRK2, MAP4K4, MAP4K5, Mapk13, METTL1, MFN2, MTCH1, MYC, MYCN, NAA40, NBR1, NDN, NEK8, NFATC2, NFYC, NPHP1, NPHP4, NR2C2, nsp15, NTRK1, OFD1, OPTN, OTUD4, OTUD5, PARP1, PATL1, PEA15, PEBP1, PEX14, PHLPP1, PIH1D1, PINK1, PLEKHA4, POLR1D, PPEF2, PPP6R2, PRKCA, PTGES3, PTK2, PTPN3, PXMP2, PYHIN1, RAB8A, RECQL4, RHOBTB1, RHOBTB2, RHOG, RND2, Rnf183, RNF41, RPAP3, RPL7A, RPTOR, RUVBL2, S100A6, SAMM50, SIAH1, SIRT6, SKP1, SLC25A12, SLC9A3R2, SNCA, SNX27, SNX4, SQSTM1, STK3, SUGT1, SYMPK, TBL1X, TP53BP1, TUBA4A, TUBB, TUBG1, UFL1, USP13, VCAM1, WWOX, ZRANB1
MYD88	ATG5, BANK1, BST2, BTK, CISH, CYLD, CYTH2, FLII, HDAC6, IL1RL2, IRAK1, IRAK2, IRAK3, IRAK4, IRF5, IRF7, LRRFIP1, LRRFIP2, MAP3K7, MYD88, PFKL, POLR1C, PRDX1, RAC1, RNF152, SASH1, SIAH1, SIAH2, SMAD3, SMAD6, SMURF1, SPOP, SYK, TIRAP, TLR2, TLR4, TLR5, TLR7, TLR9, TNIP1, TRAF3, TRAF6, TSG101, TXN, UBAP1, USP7

### CacyBP promotes Myd88 stability by blocking its interaction with Siah-1

**Fig. 3:**
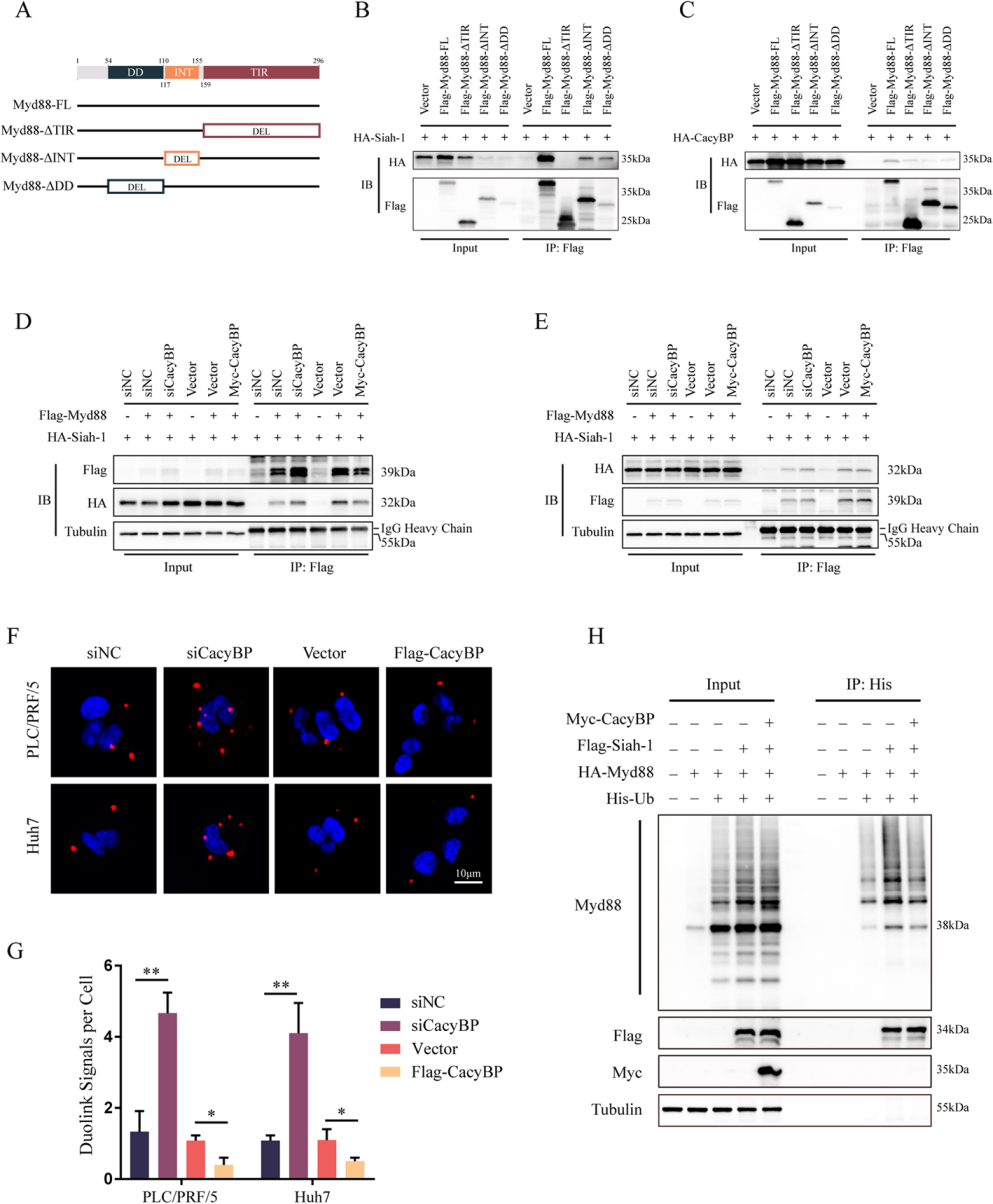
CacyBP reduces Myd88 degradation by competing and blocking its interaction with Siah-1. **A** Illustration of serial Myd88 truncation mutants. **B** Immunoprecipitation assays of exogenous Siah-1 and Myd88 truncations. **C** Immunoprecipitation assays of exogenous CacyBP and Myd88 truncations. **D**-**E** Immunoprecipitation assays of Siah-1 and Myd88 after CacyBP depletion or overexpression in PLC/PRF/5 (**D**) or Huh7 (**E**) cells. **F** Representative PLA-immunofluorescence images showing the colocation of Myd88 and Siah-1 after CacyBP silencing or overexpression in PLC/PRF/5 and Huh7 cells. **G** Quantification of the Duolink signal in (**F**). **H** Detection of Myd88 polyubiquitination in the presence of Siah-1 overexpression or Siah-1/CacyBP cooverexpression in HCC cells. *p < 0.05; **p < 0.01

To further explore the structural and functional basis of the Siah-1/Myd88 complex, we constructed a series of Flag-tagged Myd88 truncation mutants (Fig. 3A). After transient coexpression with HA-tagged CacyBP or Siah-1 in HEK293T cells, immunoprecipitation assays were performed. As shown in Fig. 3B-F, deletion of the intermediate (INT) or death domain (DD) of Myd88 partly weakened its binding to Siah-1 and the absence of the Toll/Interleukin-1 receptor (TIR) domain almost completely lost the binding ability (Fig. 3B), suggesting that the TIR domain of Myd88 was essential for its interaction with Siah-1. In addition, we also examined the binding ability of CacyBP to the Myd88 mutants and found that truncations of any domain in Myd88 significantly diminished their interaction (Fig. 3C), which indicated that CacyBP might compete with Siah-1 for Myd88 interaction. Indeed, knockdown of CacyBP enhanced the binding of Myd88 to Siah-1, while overexpression of CacyBP suppressed their interaction (Fig. 3D and E). These results were also confirmed by the proximity ligation immunofluorescence assays showing that CacyBP depletion significantly increased the dotted signals of the Siah-1 and Myd88 interaction and vice versa (Fig. 3F and G). Moreover, Siah-1 markedly increased the ubiquitination of Myd88, and coexpression of CacyBP reduced this effect (Fig. 3H). Taken together, these results suggested that CacyBP competitively interacted with Myd88 and thus protected it from Siah-1-mediated degradation.

### CacyBP-Myd88 axis inhibition reduces CX3CL1 secretion in HCC cells

Myd88-mediated signaling activates downstream NF-κB and contributes to the immunosuppressive TME in liver diseases [35]. We asked whether CacyBP had a role in regulating the activity of the NF-κB pathway. Phosphorylation status and nuclear translocation of the NF-κB subunit p65 were evaluated by western blotting and immunofluorescence in the presence of IL-1β, an agonist of Myd88 signaling, in HCC cells. After IL-1β stimulation, p65 was phosphorylated and translocated to the nucleus in HCC cells, while knockdown of CacyBP reduced p65 phosphorylation and nuclear translocation (Figure S2A and S2B). Furthermore, we examined the mRNA expression of *IL6*, *CXCL8*, and *TNF*, the three classic IL-1β-responsive genes downstream of NF-κB. However, no obvious change was observed in the transcription of these genes upon CacyBP or Myd88 inhibition, indicating that CacyBP may have effects on other targets downstream of Myd88 (Figure S2C).

**Fig. 4:**
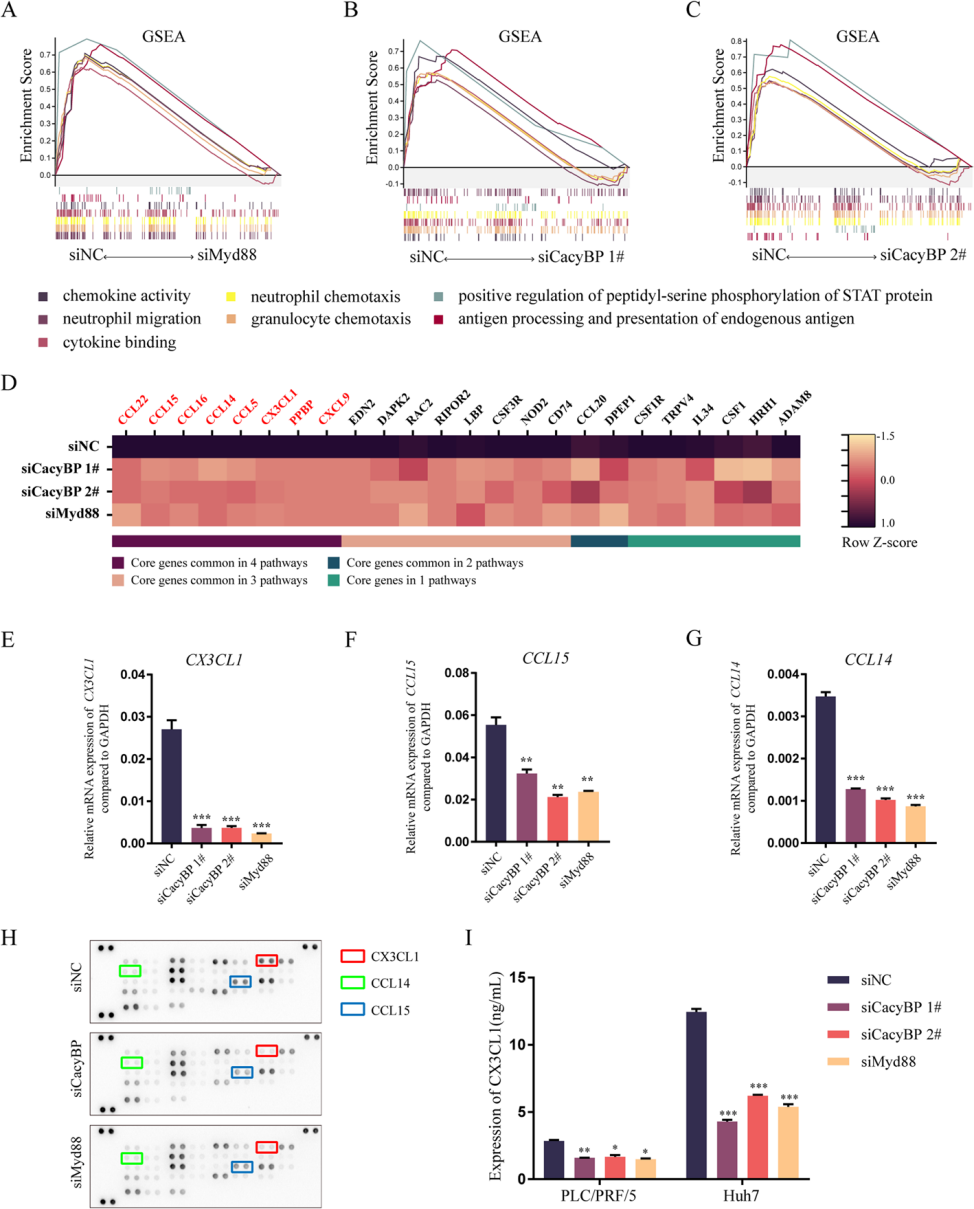
Inhibition of CacyBP-Myd88 axis decreases CX3CL1 expression and secretion in HCC cells. **A**-**C** GSEA analysis of differentially expressed genes affected by CacyBP or Myd88 depletion in HCC cells. siNC/siMyd88 (**A**), siNC/siCacyBP 1# (**B**), siNC/siCacyBP 2# (**C**). **D** Heatmap showing the significantly altered inflammation-related genes after CacyBP or Myd88 knockdown in HCC cells. The font color of chemokines is in red. **E**–**G** mRNA expression of *CX3CL1* (E), *CCL15* (**F**) or *CCL14* (**G**) after CacyBP or Myd88 depletion in HCC cells. **H** Expression of cytokine profiles in the supernatant of HCC cells after CacyBP or Myd88 knockdown. **I** ELISA analysis showing the CX3CL1 concentration in the media of HCC cells after CacyBP or Myd88 depletion. *p < 0.05; **p < 0.01; ***p < 0.001

Therefore, we performed next-generation sequencing to analyze gene expression profiling after siRNA-mediated knockdown of CacyBP or Myd88 expression in PLC/PRF/5 cells. Gene set enrichment analysis (GSEA) showed that 12 pathways were enriched among downregulated genes in both siCacyBP and siMyd88 groups, most of which were associated with immune cell migration and cytokine release (Figure S3A and Fig. 4A-C). Notably, the expression levels of some chemokine genes were consistently decreased after inhibition of CacyBP and Myd88 (Fig. 4D), and we verified these alterations by qRT-PCR and confirmed that, compared to the control group, the mRNA expression levels of *CX3CL1*, *CCL14* and *CCL15* were significantly reduced after silencing CacyBP and Myd88 in HCC cells (Fig. 4E-4 and S3B-S3F). Additionally, a human chemokine array was adopted to further investigate the differential expression of common chemotactic and inflammatory cytokines in the cell culture supernatant between groups (Fig. 4H). Based on the above assays, we identified that *CX3CL1* was the most consistent and largely downregulated gene after CacyBP or Myd88 depletion. Accordingly, knockdown of CacyBP obviously decreased its secretory level in the supernatant (Fig. 4I). Taken together, these data indicated that the CacyBP-Myd88 axis was necessary for CX3CL1 secretion in HCC cells.

### CacyBP-Myd88 axis regulates HDAC1-mediated histone deacetylation of CX3CL1 promoter

**Fig. 5:**
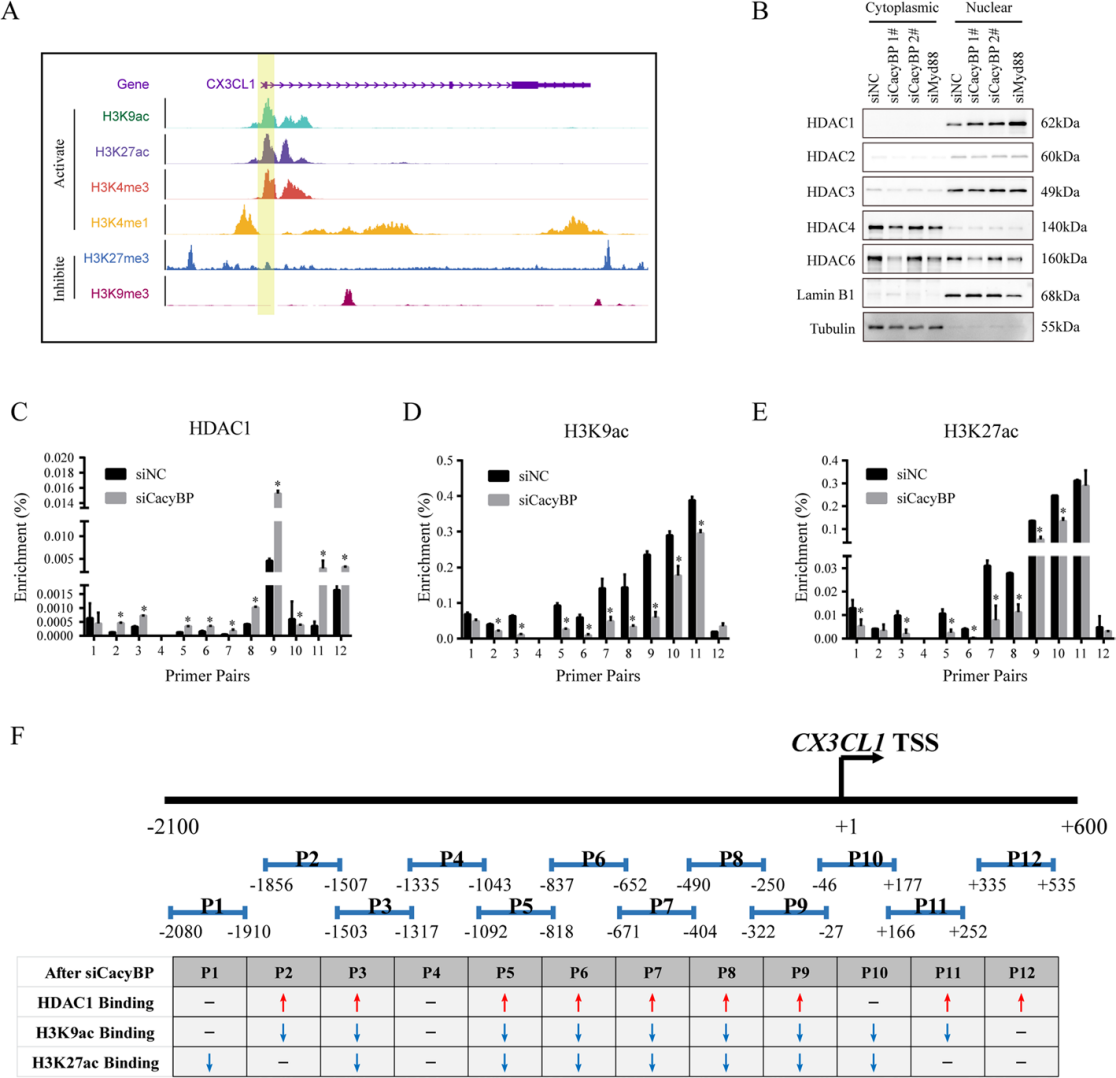
CacyBP depletion enhances HDAC1-mediated histone H3 deacetylation at *CX3CL1* promoter. **A** ChIP-seq data of HepG2 cells from ENCODE database showing the enrichment of different histone H3 modifications at *CX3CL1* gene region. **B** The nuclear and cytoplasmic expression of HDAC family proteins after CacyBP or Myd88 depletion in HCC cells. **C**-**E** The levels of HDAC1 (**C**), H3K9ac (**D**) or H3K27ac (**E**) enriched at *CX3CL1* promoter were measured by ChIP assays. **F** Statistics summary for the ChIP-PCR experiments in (C-E). Red upward arrow, increased; blue downward arrow, decreased. *p < 0.05

It has been reported that covalent histone modifications exert a profound impact on the transcriptional regulation of chemotactic cytokines, which led us to analyze the histone chromatin immunoprecipitation (ChIP)-seq data from the Encyclopedia of DNA Elements (ENCODE) database and investigate the histone modifications at *CX3CL1* promoter region. Accordingly, we found that histone markers associated with active transcription, such as H3K4me3, H3K4me1, H3K9ac and H3K27ac, were highly enriched at *CX3CL1* promoter, whereas the transcription repressive markers, H3K27me3 and H3K9me3, were much more diffusely distributed (Fig. 5A). HDACs are well described as negative regulators of histone acetylation and the crosstalk between HDACs and Myd88 signaling has also plays an important role in tumor progression [36]. Therefore, we further analyzed the nuclear and cytoplasmic expression of HDAC family proteins using western blotting. It is worth noting that only the nuclear expression of HDAC1 was increased after CacyBP or MyD88 inhibition (Fig. 5B). In addition, Myd88 depletion also increased the mRNA expression level of HDAC1 and reduced its protein degradation (Figure S4A-S4C). The results of ChIP assays consistently revealed that CacyBP knockdown enhanced the enrichment of HDAC1, but decreased the modifications of acetylated H3K9 and H3K27 at *CX3CL1* promoter in HCC cells (Fig. 5C-F). Collectively, these results indicated that inhibition of CacyBP-Myd88 axis induced HDAC1-mediated histone deacetylation of *CX3CL1* promoter.

### CacyBP-induced CX3CL1 is required for the infiltration of TAMs in HCC

**Fig. 6:**
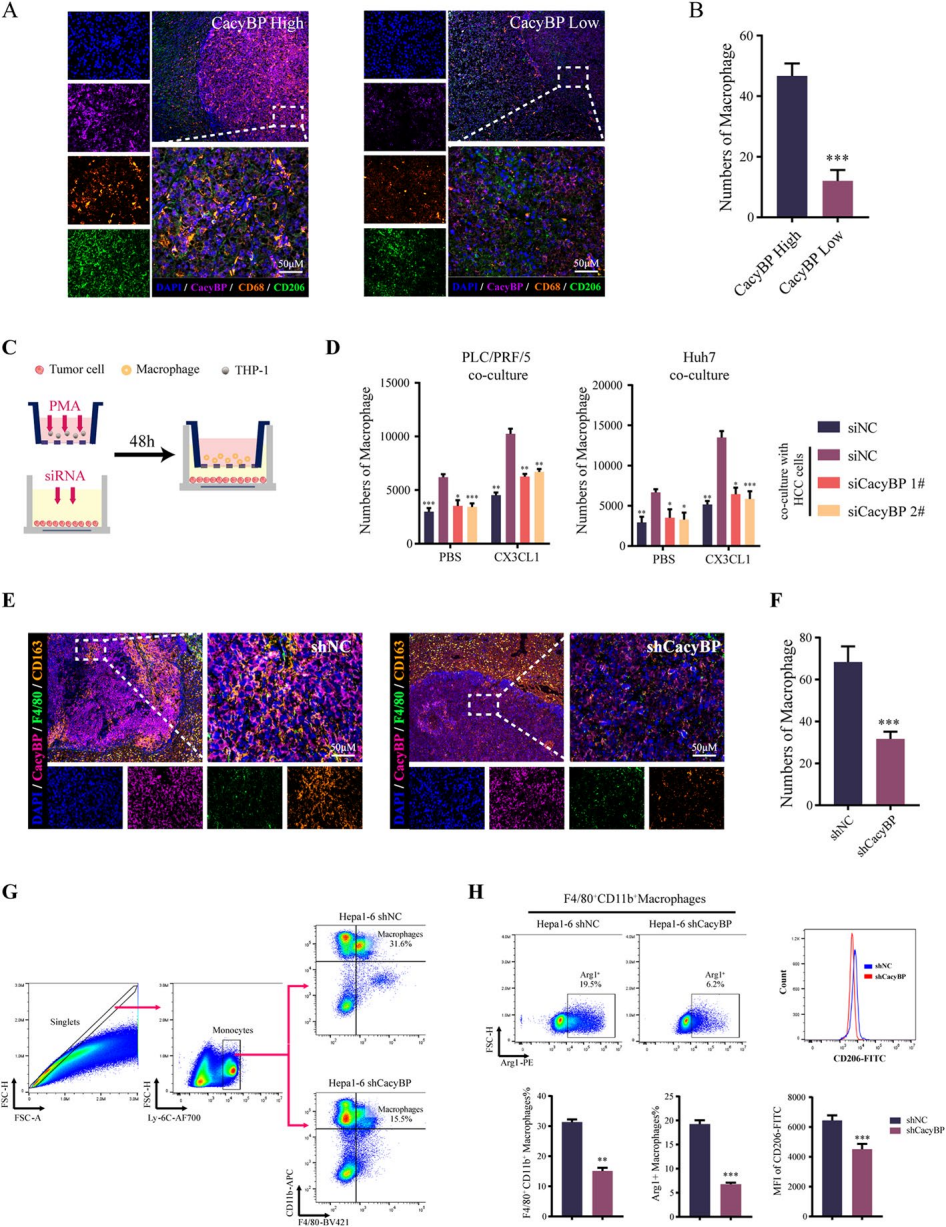
CacyBP expression is associated with macrophage recruitment in HCC. **A**-**B** Multiplex immunohistochemistry staining (**A**) and quantification (**B**) showing the infiltration of CD68^+^/CD206^+^ M2 macrophages in CacyBP high- (n = 17) or low-expressed (n = 17) HCC samples. **C** Illustration diagram showing the HCC cells cocultured with THP-1 differentiated macrophages using the 0.4-μm pore size transwell system. **D** Quantification of the number of migrated macrophages in the coculture system. **E**–**H** Multiplex immunohistochemistry staining (**E**) and quantification (**F**) showing the infiltration of F4/80^+^CD163^+^ M2 macrophages in tumor tissues formed by CacyBP-depleted or control Hepa1-6 cells from C57BL mice. **G**-**H** Flow cytometry analysis showing the proportions of infiltrating TAMs and the expression of M2 macrophage markers in tumors formed by CacyBP-depleted or control Hepa1-6 cells from C57BL mice. *p < 0.05; **p < 0.01; ***p < 0.001

As the only receptor of CX3CL1, CX3CR1 is widely expressed on immune cells, especially the peripheral monocytes. The activation of CX3CR1/CX3CL1 signaling has been identified as an essential molecular event for recruiting monocyte-derived macrophages into the hepatic microenvironment during liver inflammation and HCC progression [37, 38]. Hence, we sought to examine the effect of CacyBP deficiency on the infiltration of TAMs in HCC. We utilized the TIMER database to explore the correlations between gene expression and the degree of TAM infiltration, and the results illustrated that CacyBP, CX3CL1 and Myd88 expression was appreciably positively correlated with the infiltration levels of total macrophages and M2-type macrophages (Figure S5A-S5C). We further verified in HCC tissue slices that patients with high CacyBP expression showed an increased degree of CD68^+^CD206^+^ M2 macrophage infiltration compared with those with low CacyBP levels (Fig. 6A-B). Hepatic TAMs can be derived from in situ proliferation of tissue-resident Kupffer cells or differentiation of newly-recruited peripheral blood monocytes. We used condition medium from CacyBP or Myd88-silenced HCC cells to polarize PMA-stimulated THP-1 monocytes to the M2 macrophages, simulating the differentiation process of the tissue-resident macrophages, and flow cytometry analysis showed that interfering with CacyBP or Myd88 did not affect the expression of CD11b and CD206, two typical markers for identifying M2 macrophage differentiation (Figure S5D-S5E), indicating that CacyBP may otherwise increase the number of TAMs in a manner by promoting the recruitment of peripheral monocytes. Additionally, in the coculture system, blockade of CacyBP in HCC cells significantly reduced the chemotaxis of THP-1 cells in the upper chamber, and this effect can be rescued by adding exogenous CX3CL1 to the supernatant (Figure S5F-S5G and Fig. 6D). Moreover, we established an orthotopic liver tumor model by injecting either shCacyBP or shNC Hepa1-6 cells and the in situ formed liver tumors were then isolated for analysis (Figure S5H-S5J). The results from multiplex immunohistochemistry showed that tumors generated by CacyBP-depleted Hepa1-6 cells displayed an obvious reduction in infiltrated F4/80^+^CD163^+^ M2 macrophages (Fig. 6E and F). Consistently, flow cytometry analysis revealed that the fraction of TAMs was significantly decreased in CacyBP-depleted liver tumors. Specifically, these TAMs also exhibited diminished expression of M2 macrophage markers, such as CD68, CD206 and arginase-1 (Fig. 6G and H). Taken together, the above results indicated that CacyBP inhibition markedly reduced the infiltration of TAMs in HCC.

### Blockade of CacyBP enhances the therapeutic efficacy of anti-PD-1 antibody in HCC

**Fig. 7:**
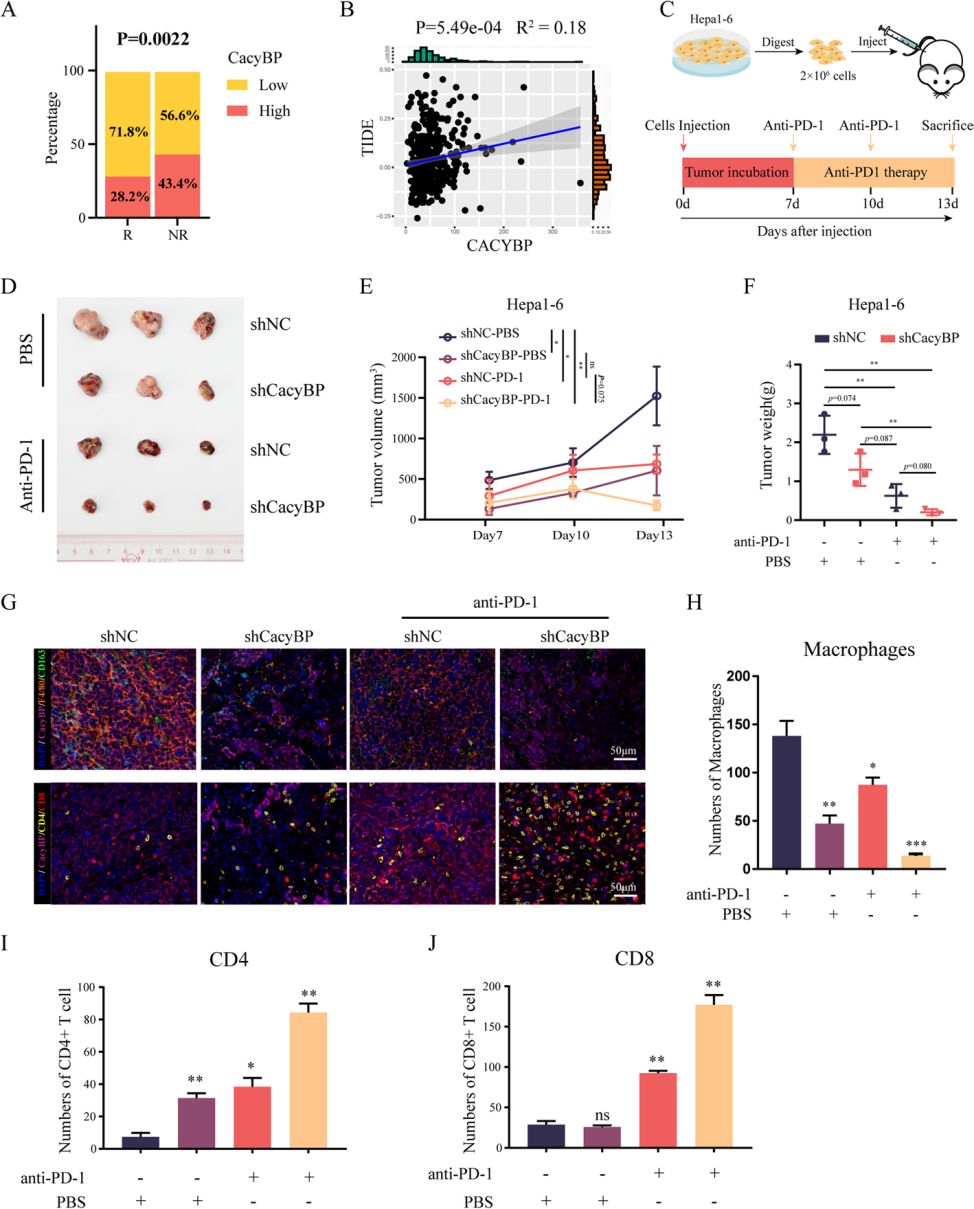
Inhibition of CacyBP-Myd88 axis enhances the therapeutic efficacy of anti-PD-1 antibodies in HCC. **A** Comparison of CacyBP expression scores between predicted anti-PD-1 responder and nonresponder tumors, as determined by the TIDE algorithm. **B** Correlation between TIDE score and CacyBP expression. **C** Schematic illustration of the anti-PD-1 therapy survey experiments in a mouse HCC tumor model. **D** Xenograft tumors isolated from C57BL mice are shown. **E**–**F** Growth curves (**E**) and weights (**F**) of xenograft tumors from C57BL mice injected with control and CacyBP-depleted Hepa1-6 cells with or without anti-PD-1 treatment. **G** Multiplex immunohistochemistry staining showing the infiltration of F4/80^+^CD163^+^ M2 macrophages, CD4^+^ and CD8^+^ T cells in tumors formed by CacyBP-depleted or control Hepa1-6 cells from C57BL mice. **H**-**J** Quantification of the number of infiltrated macrophages (**H**), CD4^+^ (**I**) and CD8^+^ (**J**) T cells in (**G**).ns, not significant; *p < 0.05; **p < 0.01; ***p < 0.001

TAM infiltration has been known to play a role in inducing immune tolerance and anti-PD-1 therapy resistance. To further explore whether CacyBP inhibition can improve the therapeutic effect of immunotherapy, we used the TIDE algorithm [39] to predict the correlation between CacyBP expression and the anti-PD-1 response in HCC patients. HCC patients in the low-CacyBP group presented a lower TIDE score (Fig. 7A and B), implying that these patients may be more sensitive to anti-PD-1 therapy. Next, we established a xenograft tumor model by subcutaneous implantation of either shNC or shCacyBP Hepa1-6 cells in C57BL mice, and the mice were subjected to anti-PD-1 or control isotype antibody administration, respectively. In consistence with our previous findings, inhibition of CacyBP impaired tumor growth and decreased tumor infiltration of F4/80^+^CD163^+^ macrophages (Fig. 7C-F). Meanwhile, the superposition of anti-PD-1 antibody not only further suppressed the tumor growth and macrophage infiltration, but also restored the antitumor activity of resident T cells (Fig. 7G-J). Notably, among all of the groups, the combination of shCacyBP plus anti-PD1 treatment achieved an optimal anti-HCC efficacy. Collectively, these results demonstrated that CacyBP has the potential to be an important target for sensitizing HCC to anti-PD-1 immunotherapy.

## Discussion

In the present study, we demonstrated that high expression of CacyBP in HCC was correlated with anti-PD-1 tolerance. Specifically, we identified that Myd88 as a novel binding substrate of CacyBP, that directly interacts with the TIR region of Myd88 and prevents it from proteasomal degradation by the E3 ubiquitin ligase Siah-1. The sustained expression of Myd88 inhibited HDAC1-mediated histone deacetylation of *CX3CL1* promoter, thereby promoting CX3CL1 transcriptional activation and secretion. This process further increased the recruitment of TAMs to the HCC microenvironment, inducing immune escape and anti-PD-1 tolerance (Figure S6). Therefore, our study indicated that CacyBP may be a promising target for anti-PD-1 therapy in HCC.

The majority of HCC patients develop on the background of chronic inflammation, activating a series of cellular processes and oncogenic signaling pathways to enhance HCC development [40]. It is well known that Myd88 is a central node of inflammatory pathways, which links the IL-1/TLR and Ras oncogenic signaling pathways for the induction of proinflammatory cytokines and the maintenance of tumor cell viability [41]. Overexpression of Myd88 has been found to accelerate tumor progression and is correlated with the poor clinical outcomes in various types of cancers, including HCC. For example, mice lacking Myd88 displayed a reduction in hepatocarcinogenesis in DEN-induced liver cancer [42]. Consistently, IL-6 is one of the major inflammatory mediators that stimulates HCC development, and Myd88 deficiency prevents HBx-mediated IL-6 secretion [43]. Furthermore, Myd88 signaling also contributed to promoting HCC development via upregulated IL-23/IL-17A expression [44]. Thus, activation of Myd88 signaling was required for HCC development and progression. In the current study, we found that CacyBP can act as an upstream regulator of Myd88. In HCC specimens, CacyBP expression was significantly associated with Myd88. Knockdown of CacyBP in HCC cell lines reduced the protein level of Myd88 by Siah-1-mediated proteasomal degradation, while enforced expression of CacyBP had the opposite effect. Indeed, Myd88 was revealed to be mainly degraded through the ubiquitin–proteasome system, and a number of E3 ligases have been identified to modulate Myd88 expression [45–48]. Among them, the literature has pointed out that Siah-1 induced by DENV2 infection could serve as a potential binding partner of Myd88 [49]. Consistently, we also confirmed their interaction in HCC cell lines, and overexpression of Siah-1 markedly shortened the half-life of Myd88 and induced its polyubiquitination and subsequent degradation, which can be blocked by the proteasome inhibitor MG132. By competitively binding to the TIR domain of Myd88, CacyBP can thereby prevent it from Siah-1-induced degradation. This may be one of the important reasons for the sustained high expression of Myd88 in HCC.

Upon signal activation, the TIR domain of Myd88 mediates the interaction with intracellular TIR domains of the TLRs, while DD recruits IRAK4 and IRAK1/2 to form a myddosome complex and activates the NF-κB pathway through the phosphorylation cascade. As the first step in this process, TIR domain-mediated dimerization is essential for signal initiation, and overactivation of TIR domain-mediated signaling has been found in many inflammatory diseases and tumor progression [50]. It should be pointed out that the TIR domain appears to be more readily interfered by some negative regulators, which competes with other binding partners and modifies the ubiquitination states of Myd88 for proteasomal degradation [51]. Consistent with this notion, we demonstrated that the integrity of the TIR domain is critical for Siah-1 and CacyBP binding to Myd88, however, the specific structural basis for their competitive interaction remains to be elucidated.

A previous study has demonstrated that HDAC1 was rapidly degraded when cells were treated with the proinflammatory cytokine TNF-α, which required IKK2 activity downstream of Myd88 signaling [52]. In fact, HDAC1 depletion may be a feedback of NF-κB signaling activation. In that situation, the cells rapidly transcribe a large number of proinflammatory genes and the ubiquitously expressed HDAC1 is not beneficial to the transcription events since it can repress the transcription of proinflammatory genes by deacetylating histones and promoting chromatin compaction [53–55]. Thus, the degradation of HDAC1 by proinflammatory stimuli may provide a suitable environment for the initiation of subsequent transcription events. In our study, we demonstrated that Myd88 expression is essential for the mRNA expression and protein stability of HDAC1, indicating that CacyBP-Myd88 axis relieves the transcriptional inhibitory signal of proinflammatory cytokines in HCC cells by regulating histone modification enzymes.

CX3CL1 is the only member of the CX3C chemokine family and is constitutively expressed in a variety of cells, including epithelial tumor cells. The two forms of CX3CL1 may exert different biological functions. Membrane-attached mCX3CL1 promotes the adhesion of leucocytes to endothelial cells under physiological flow conditions, while, the soluble sCX3CL1, produced after the cleavage by several metalloproteases, acts mainly as a potent chemoattractant targeting on its receptor CX3CR1 expressed on most immune cells, especially the peripheral monocytes [56, 57]. TAMs derived from the peripheral monocytes account for the most abundant immune cell type in the TME. Generally, proinflammatory M1 macrophages may present with low CX3CR1 expression, and these cells are associated with longer OS for cancer patients, whereas, proinflammatory M2 macrophages or TAMs contain a higher CX3CR1 expression, and an increased percentage of these cells leads to a worse clinical outcome [58, 59]. CX3CL1/CX3CR1 signaling is constantly activated in the process of monocyte recruitment and subsequent differentiation to TAMs in the tumor niche [60]. In addition, CX3CL1 increased the secretion of platelet-derived factor 4 (PDF-4)/CXCL4 in macrophages and cooperated with MMP9 released by stromal cells to support tumor angiogenesis, which further accelerated the accumulation of monocytes from peripheral blood [61, 62]. Thus, blockade of the CX3CL1/CX3CR1 axis may contribute to the enhancement of antitumor therapy by reversing the inhibitory immune environment. In our study, we demonstrated inhibition of CacyBP/Myd88 signaling markedly reduced the CX3CL1-mediated chemotaxis of monocytes and macrophages, and increased the infiltration of CD4^+^ and CD8^+^ T cells in the TME, which provides a novel idea for improving therapeutic efficacy of anti-PD-1 antibodies in HCC.

Systemic treatment of advanced HCC is constantly evolving, and a variety of first and second line therapies including tyrosine kinase inhibitors (TKIs) and ICIs are now available for improving prognosis. These agents exert antitumor effects by inhibiting molecular pathways that are crucial for tumor growth or promoting tumor immunosuppressive microenvironments. The combination of ICIs plus TKIs targets not only the tumor itself but also the microenvironment, potentially synergizing the treatment process. A number of studies, including our own one [32], have identified that CacyBP as an independent risk factor for HCC and it is critical for tumor growth and proliferation [27–30]. In current study, we demonstrated that knockdown of CacyBP reduces Myd88-mediated CX3CL1 secretion and subsequent infiltration of TAMs, weakening the immunosuppressive state of TME. Therefore, targeting CacyBP can not only inhibit the growth of tumor itself, but also extend the current approaches of anti-PD-1-based immunotherapy.

### Supplementary Information

**Additional file 1:** **Figure S1.** Siah-1 is identified as a binding partner of both Myd88 and CacyBP. (A) Venn diagram displaying the interacting proteins of CacyBP and Myd88 in the BioGrid database. (B-C) Immunoprecipitation assays of HEK293T cells expressing HA-tagged CYLD (B) or Siah-1 (C) and Flag-tagged CacyBP. (D) Exogenous Flag-tagged Myd88 was degraded by HA-tagged Siah-1, but not HA-tagged CYLD in HEK293T cells. (E) Representative immunohistochemistry images of Siah-1 expression from 34 HCC tissues and their matched adjacent nontumor tissues. T, tumor; N, nontumor. (F) Quantification of Siah-1 expression scores in tumor tissues and nontumor tissues from 34 HCC slices. (G) Representative images of high and low Siah-1 expression in HCC tissues. (H-I) Siah-1 expression was significantly associated with OS (I) but not with DFS (J) in our HCC cohort according to Kaplan-Meier analysis. Median OS: Siah-1 High (Undefined), Siah-1 Low (38.2 months); Median DFS: Siah-1 High (Undefined), Siah-1 Low (38.1 months). ***p < 0.001.

**Additional file 2:** **Figure S2.** Knockdown of CacyBP or Myd88 inhibits the activation of NF-κB pathway. (A) Representative immunofluorescence images of NF-κB subunit p65 in HCC cells treated as indicated. Bar: 10 μm. (B) Detection of phospho-p65 and Myd88 expression in HCC cells treated as indicated. (C) The mRNA expression levels of*IL6*, *CXCL8* and *TNF* in HCC cells treated as indicated. ns, not significant; *p < 0.05;***p < 0.001.

**Additional file 3:** **Figure S3.** Chemokine expression is significantly altered in PLC/PRF/5 cells after CacyBP and Myd88 depletion. (A) Venn diagram showing the common enriched pathways as indicated. (B-F) The mRNA expression levels of *CCL22*, *CXCL9*, *PPBP*, *CCL16* and *CCL5* were verified by qPCR in HCC cells after CacyBP or Myd88 depletion. ns, not significant; **p < 0.01; ***p < 0.001.

**Additional file 4:** **Figure S4.** Knockdown of CacyBP or Myd88 increases HDAC1 expression in HCC cells. (A) Detection of *HDAC1* mRNA expression in HCC cells after CacyBP or Myd88 depletion. (B) CHX chase assay of HDAC1 protein in HCC cells after Myd88 depletion. (C) HDAC1 protein degradation curve in HCC cells after Myd88 depletion. *p< 0.05; **p < 0.01.

**Additional file 5:** **Figure S5.** CacyBP expression is associated with macrophage recruitment in HCC. (A-C) Correlation analysis between CacyBP (A), Myd88 (B) or CX3CL1 (C) expression adn the infiltration degree of total macrophages or M2 macrophages in LIHC cohort from the TCGA database. (D-E) CD206 expression in HCC cells after CacyBP deletion detected by flow cytometry. (F-G) Representative images of migrated THP-1 differentiated macrophages cocultured with HCC cells. Bar: 100 μm. (H) The livers were isolated from C57BL mice after orthotopic injection of shNC or shCacyBP Hepa1-6 cells. The yellow dashed line depicts the area of the tumor. (I) CacyBP protein levels were verified in the orthotopic liver tumors. (J) HE staining confirmed tumor formation in the liver in (H). Bar: 100 μm. ns, not significant; **p < 0.01; ***p < 0.001.

**Additional file 6:** **Figure S6.** Schematic diagram depicting the molecular mechanism of CacyBP/Myd88 axis-driven TAMs recruitment.

## Data Availability

All data supporting the findings of this study are available from the corresponding authors upon reasonable request.
