# Supplementary figures and images for: Blockage of CacyBP inhibits macrophage recruitment and improves anti-PD-1 therapy in hepatocellular carcinoma
Source: J Exp Clin Cancer Res. 2023 Nov 16;42:303. doi: 10.1186/s13046-023-02885-w (PMC10652496; doi:10.1186/s13046-023-02885-w)

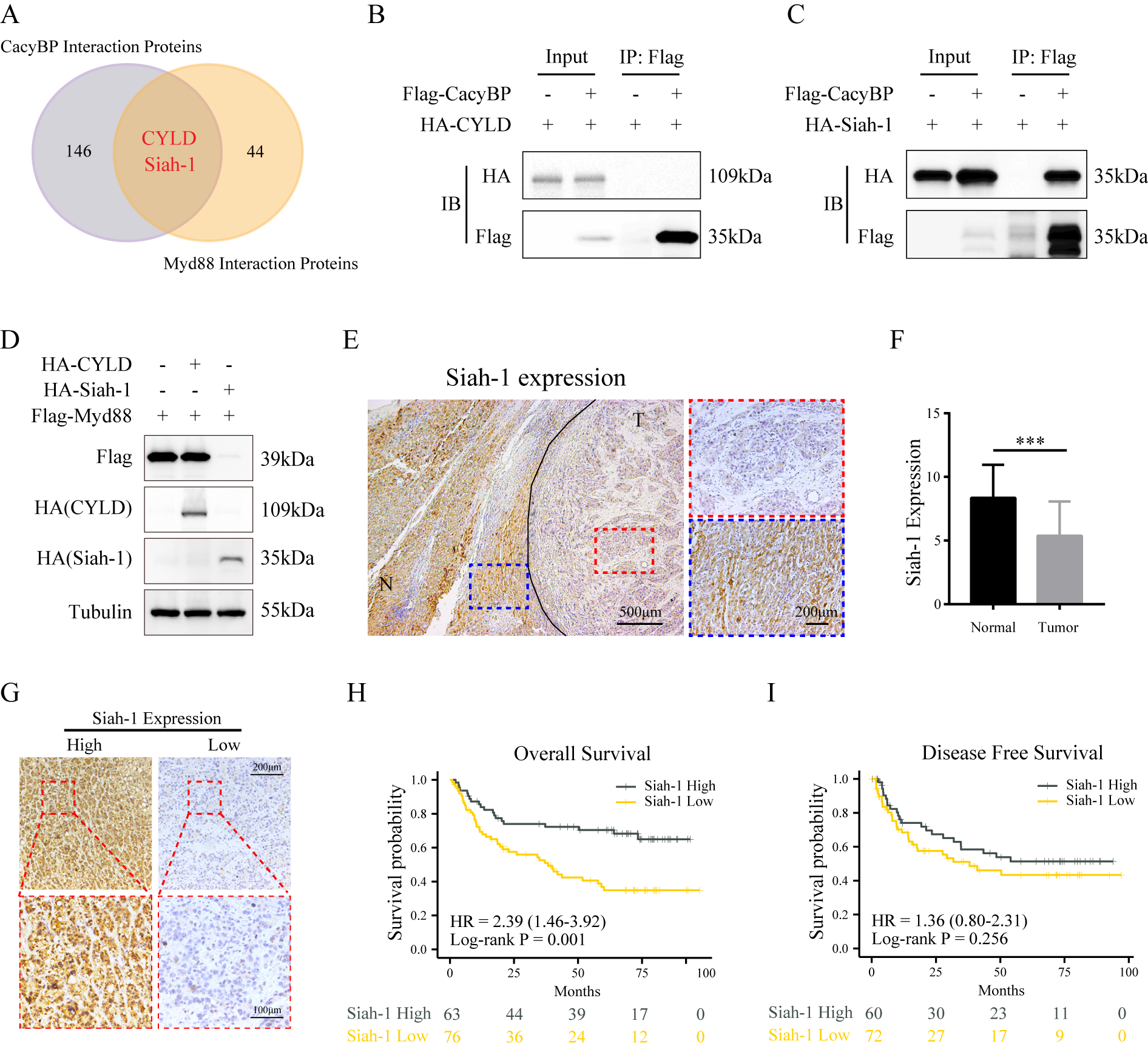

Supplement: Supplementary file 1 — Additional file 1: Figure S1. Siah-1 is identified as a binding partner of both Myd88 and CacyBP. (A) Venn diagram displaying the interacting proteins of CacyBP and Myd88 in the BioGrid database. (B-C) Immunoprecipitation assays of HEK293T cells expressing HA-tagged CYLD (B) or Siah-1 (C) and Flag-tagged CacyBP. (D) Exogenous Flag-tagged Myd88 was degraded by HA-tagged Siah-1, but not HA-tagged CYLD in HEK293T cells. (E) Representative immunohistochemistry images of Siah-1 expression from 34 HCC tissues and their matched adjacent nontumor tissues. T, tumor; N, nontumor. (F) Quantification of Siah-1 expression scores in tumor tissues and nontumor tissues from 34 HCC slices. (G) Representative images of high and low Siah-1 expression in HCC tissues. (H-I) Siah-1 expression was significantly associated with OS (I) but not with DFS (J) in our HCC cohort according to Kaplan-Meier analysis. Median OS: Siah-1 High (Undefined), Siah-1 Low (38.2 months); Median DFS: Siah-1 High (Undefined), Siah-1 Low (38.1 months). ***p < 0.001. [file 13046_2023_2885_MOESM1_ESM.tif]

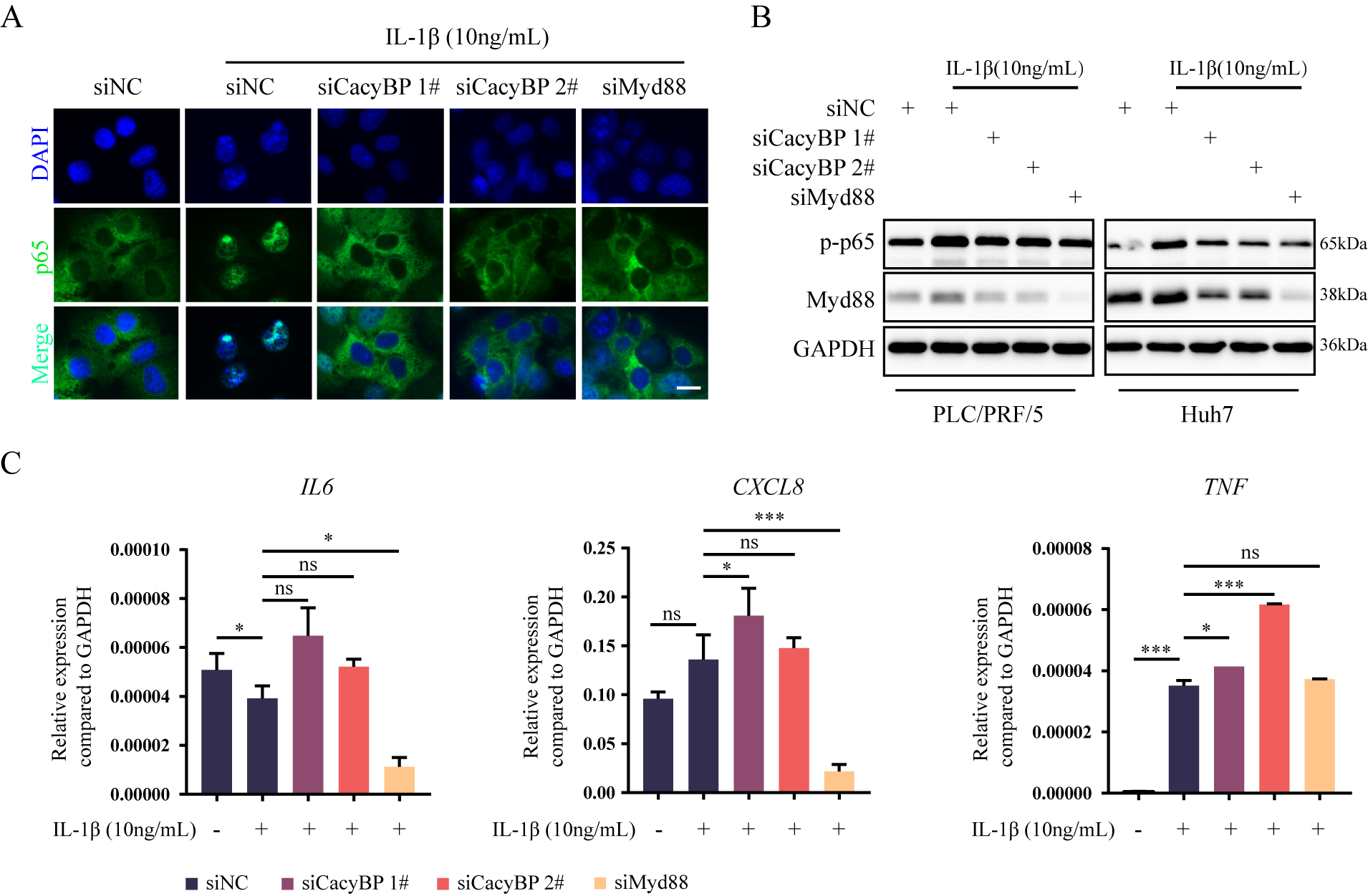

Supplement: Supplementary file 2 — Additional file 2: Figure S2. Knockdown of CacyBP or Myd88 inhibits the activation of NF-κB pathway. (A) Representative immunofluorescence images of NF-κB subunit p65 in HCC cells treated as indicated. Bar: 10 μm. (B) Detection of phospho-p65 and Myd88 expression in HCC cells treated as indicated. (C) The mRNA expression levels ofIL6, CXCL8 and TNF in HCC cells treated as indicated. ns, not significant; *p < 0.05;***p < 0.001. [file 13046_2023_2885_MOESM2_ESM.tif]

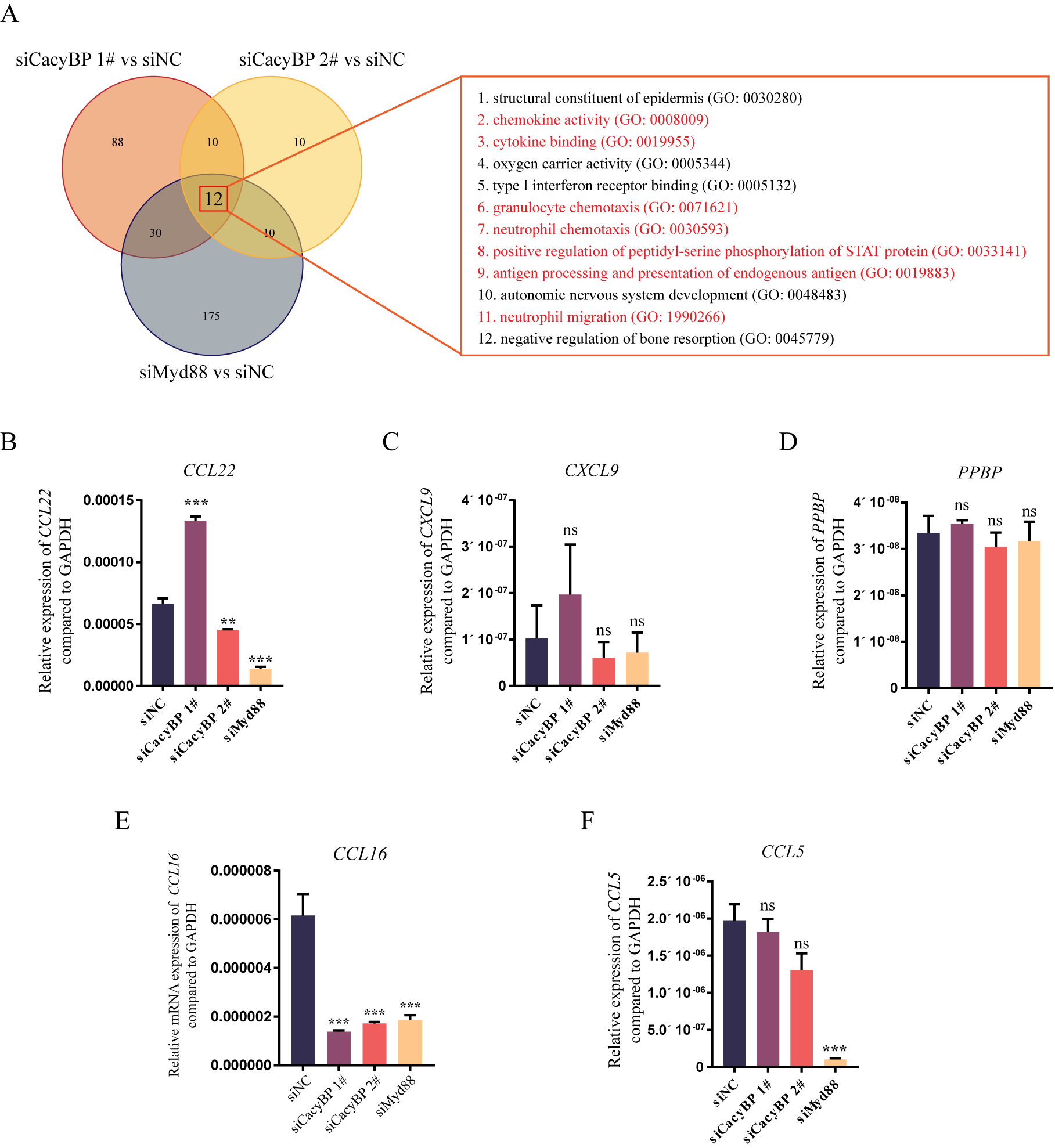

Supplement: Supplementary file 3 — Additional file 3: Figure S3. Chemokine expression is significantly altered in PLC/PRF/5 cells after CacyBP and Myd88 depletion. (A) Venn diagram showing the common enriched pathways as indicated. (B-F) The mRNA expression levels of CCL22, CXCL9, PPBP, CCL16 and CCL5 were verified by qPCR in HCC cells after CacyBP or Myd88 depletion. ns, not significant; **p < 0.01; ***p < 0.001. [file 13046_2023_2885_MOESM3_ESM.tif]

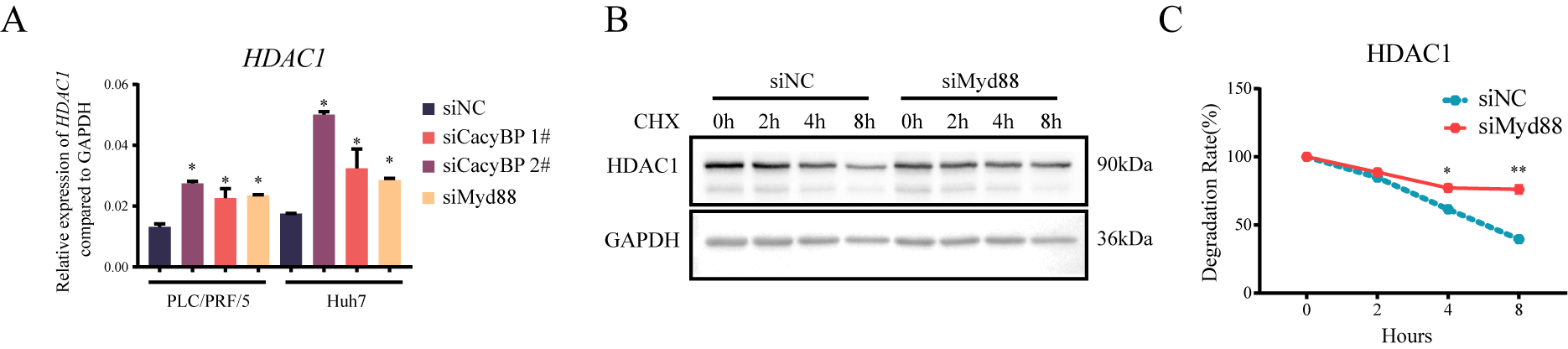

Supplement: Supplementary file 4 — Additional file 4: Figure S4. Knockdown of CacyBP or Myd88 increases HDAC1 expression in HCC cells. (A) Detection of HDAC1 mRNA expression in HCC cells after CacyBP or Myd88 depletion. (B) CHX chase assay of HDAC1 protein in HCC cells after Myd88 depletion. (C) HDAC1 protein degradation curve in HCC cells after Myd88 depletion. *p< 0.05; **p < 0.01. [file 13046_2023_2885_MOESM4_ESM.tif]

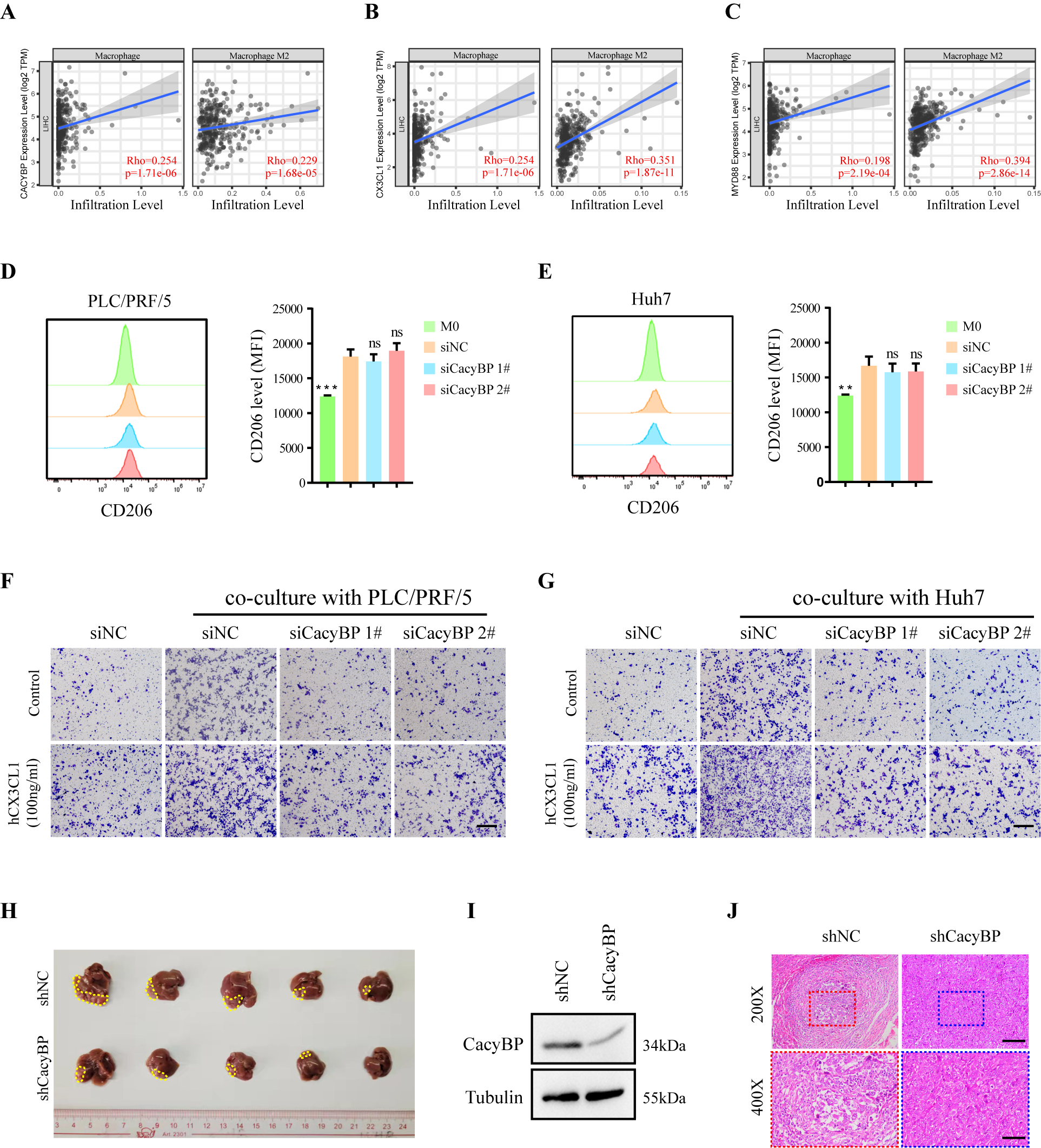

Supplement: Supplementary file 5 — Additional file 5: Figure S5. CacyBP expression is associated with macrophage recruitment in HCC. (A-C) Correlation analysis between CacyBP (A), Myd88 (B) or CX3CL1 (C) expression adn the infiltration degree of total macrophages or M2 macrophages in LIHC cohort from the TCGA database. (D-E) CD206 expression in HCC cells after CacyBP deletion detected by flow cytometry. (F-G) Representative images of migrated THP-1 differentiated macrophages cocultured with HCC cells. Bar: 100 μm. (H) The livers were isolated from C57BL mice after orthotopic injection of shNC or shCacyBP Hepa1-6 cells. The yellow dashed line depicts the area of the tumor. (I) CacyBP protein levels were verified in the orthotopic liver tumors. (J) HE staining confirmed tumor formation in the liver in (H). Bar: 100 μm. ns, not significant; **p < 0.01; ***p < 0.001. [file 13046_2023_2885_MOESM5_ESM.tif]

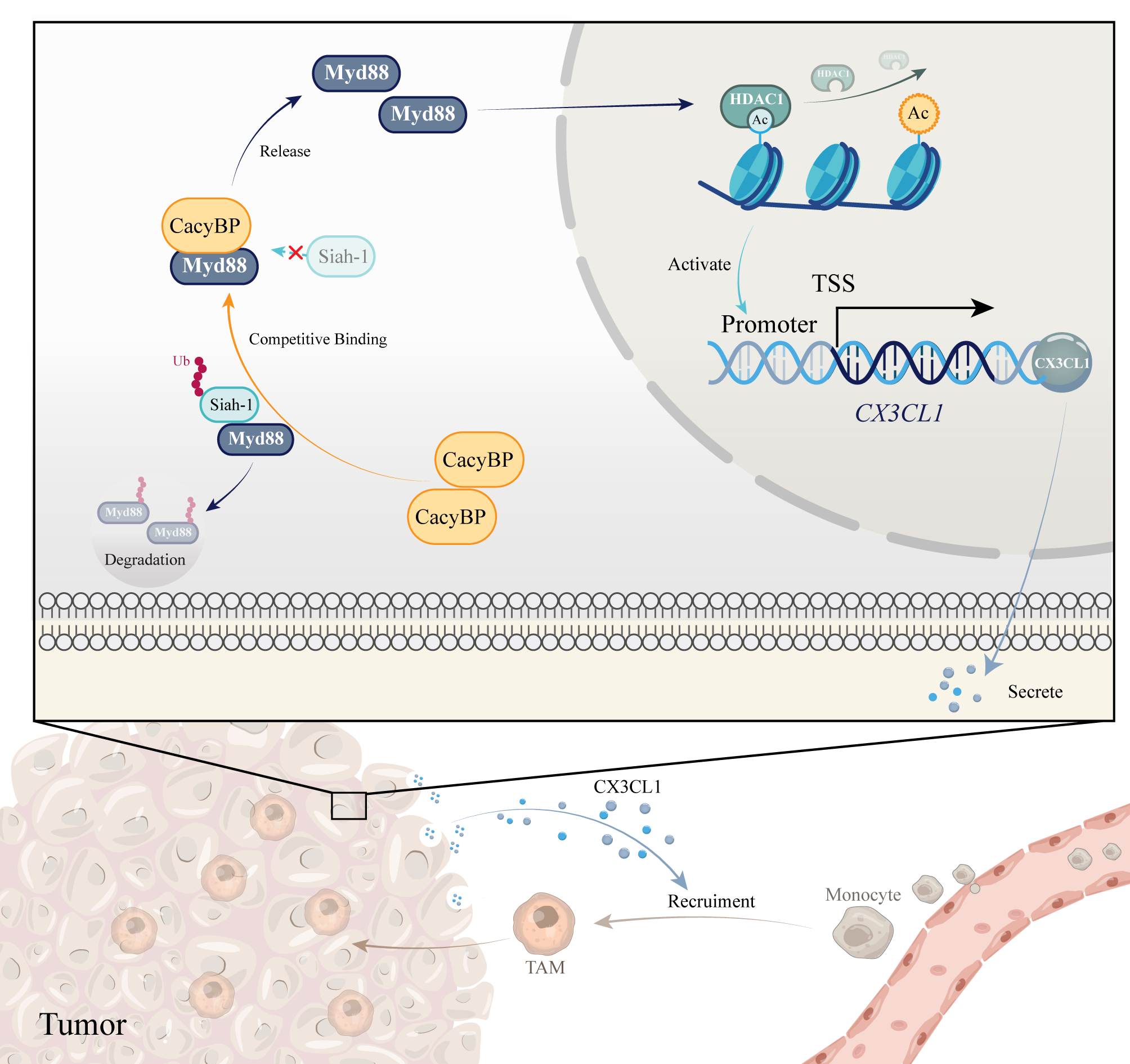

Supplement: Supplementary file 6 — Additional file 6: Figure S6. Schematic diagram depicting the molecular mechanism of CacyBP/Myd88 axis-driven TAMs recruitment. [file 13046_2023_2885_MOESM6_ESM.tif]
